# Effect of Olive Stone Biomass Ash Filler in Polylactic Acid Biocomposites on Accelerated Weathering Tests

**DOI:** 10.3390/polym18010030

**Published:** 2025-12-23

**Authors:** José Ángel Moya-Muriana, Francisco J. Navas-Martos, Sofía Jurado-Contreras, Emilia Bachino-Fagalde, M. Dolores La Rubia

**Affiliations:** 1 Andaltec Technological Centre, Ampliación Polígono Industrial Cañada de la Fuente, C/Vilches 34, 23600 Martos, Spainfrancisco-javier.navas@andaltec.org (F.J.N.-M.); sofia.jurado@andaltec.org (S.J.-C.); 2 Department of Chemical, Environmental and Materials Engineering, Campus Las Lagunillas, University of Jaén, 23071 Jaén, Spain; 3 University Institute for Research in Olive Grove and Olive Oil (INUO), Campus Las Lagunillas, University of Jaén, 23071 Jaén, Spain

**Keywords:** olive stone ash, polylactic acid, biocomposites, accelerated weathering, kinetic analysis

## Abstract

Polylactic acid (PLA) is a widely used bio-based polymer, although its application is limited by mechanical brittleness and low thermal resistance. PLA-based biocomposites reinforced with waste materials are gaining attention due to their sustainability, but their durability under degradation conditions remains a key concern. In this work, PLA biocomposites containing 0, 1, and 3% wt. of Olive-stone Biomass Ash (OBA) were manufactured and characterized both (1) after manufacture and (2) after laboratory-accelerated weathering (including UV exposure, heat, and humidity). The results obtained were analyzed to evaluate the influence of ash incorporation on degradation resistance (measured through Carbonyl Indices, CI), mechanical properties (tensile strength), thermal (Thermogravimetric Analysis—Differential Scanning Calorimetry, TGA-DSC), structure (Fourier Transform Infrared Spectroscopy, FT-IR), morphology (Scanning Electron Microscopy, SEM) and appearance (colorimetry and gloss). Key quantitative findings include a 35% reduction in tensile strength for raw PLA after 1000 h weathering exacerbated to 48% and 50% with 1% and 3% OBA incorporation, respectively. Degradation indices showed increased hydroxyl formation, with HI values ranging from 0.38 to 2.80 for PLA, while for biocomposites HI rose up to 5.85 for PLA with 3% OBA. Subsequently, a solid-state reaction was model-fitted from experimental data obtained by means of TGA analysis for determining the kinetic triplet (pre-exponential factor, the activation energy, and the reaction mechanism). Finally, the Acceleration Factor (AF), which combines the effects of radiation, temperature, and humidity to predict long-term material performance, is addressed analytically.

## 1. Introduction

In recent years, the drive towards sustainable materials has focused on polylactic acid (PLA), a biodegradable, bio-based polymer derived from renewable resources such as corn starch or sugar cane [1,2]. PLA represents a promising alternative to petroleum-based plastics, in line with the principles of the circular economy, as it offers a reduced environmental footprint thanks to its 100% renewable origin and its ability to degrade into non-toxic components under industrial composting conditions (high temperature, controlled humidity, and specific microorganisms), although its degradation in natural environments, such as soil or oceans, is significantly slower [2,3,4]. This focus on sustainability has also driven interest in the valorization of biomass waste, such as olive pit biomass ash (OBA), as a filler in PLA biocomposites. OBA, a by-product of olive oil production prevalent in Mediterranean regions, exemplifies the circular economy by reusing agricultural waste in functional materials, thus minimizing waste and resource depletion, while potentially improving PLA properties [5].

The valorization of olive industry by-products has been extensively studied in polymer composites, primarily focusing on the use of ground olive pits (olive stone flour) as lignocellulosic fillers in matrices such as polystyrene, polypropylene, and PLA [6,7,8]. These studies generally report that while olive pit powder can increase stiffness (Young’s modulus), it often reduces tensile strength due to poor interfacial adhesion [7,8]. In contrast, the application of olive pit biomass ash, the residue obtained after energetic valorisation, has been largely limited to the construction sector, where it serves as a secondary raw material for fired clay bricks [9] or as an eco-friendly cement substitute in mortar [10]. To the best of our knowledge, the incorporation of olive pit ash as a filler in polylactic acid (PLA) matrices has not been reported in the literature. This work addresses this gap by evaluating the potential of this thermal power plant by-product as a sustainable inorganic filler, specifically analyzing its effect on the mechanical and thermal performance of the biocomposite.

Despite its advantages, PLA faces a major drawback: its limited service life, especially when exposed to environmental stressors [11,12]. As a biodegradable polymer, PLA offers advantages such as low toxicity and recyclability, but its susceptibility to degradation under adverse conditions, such as solar radiation, heat, and humidity, restricts its durability in applications requiring prolonged outdoor use [13]. These factors trigger different degradation mechanisms in PLA and its biocomposites. Solar radiation, especially ultraviolet (UV) light, induces photodegradation through processes such as photo-oxidation and Norrish type I and II photolysis, which causes chain scission and reduces molecular weight, compromising mechanical properties such as tensile strength and ductility [14,15]. Heat accelerates these degradation reactions, while moisture promotes hydrolysis, breaking the ester bonds in the PLA structure and further weakening the material [16]. In biocomposites, fillers such as OBA can modify these mechanisms, potentially by shielding UV light, altering thermal stability, or influencing moisture absorption, depending on their interaction with the PLA matrix [17,18,19].

To assess the durability of PLA and its biocomposites under such conditions, accelerated weathering tests in controlled laboratory environments are used. Among these, xenon arc lamp systems are particularly effective, as they closely simulate natural cycles of sunlight, temperature, and humidity [20]. Xenon lamps reproduce the spectral distribution of sunlight, making them ideal for studying photodegradation over a condensed period of time [21]. The validity of these tests depends on the reciprocity law, which states that the total energy absorbed by the material determines the degree of degradation, regardless of the exposure rate [22]. In addition, the acceleration factor (AF) quantifies how much faster degradation occurs in the laboratory compared to natural conditions, providing a critical link between controlled experiments and practical applications [23].

The main objective of this study is to investigate the effects of incorporating olive pit biomass ash (OBA) as a reinforcing filler in PLA biocomposites on their accelerated weathering resistance. PLA biocomposites with varying OBA contents (0%, 1%, and 3% by weight) were fabricated and subjected to xenon arc weathering tests for 500 and 1000 h, with a comprehensive characterization of their mechanical, thermal, structural, morphological, and appearance properties, performed before and after exposure. The main results reveal that OBA accelerates degradation under weathering conditions, especially at high loads, due to poor interfacial adhesion and increased moisture absorption, resulting in reduced mechanical strength and increased hydrolytic decomposition. However, OBA can also act as a nucleating agent that improves the crystallinity of PLA, particularly when the particles have an appropriate size and surface characteristics, which influence thermal behavior and produce distinctive aesthetic changes, such as whitening and gloss retention, due to the combined effects of polymer degradation and the presence of filler [24]. These results highlight the advantages and disadvantages of using OBA as a sustainable filler and underline the need for further optimization to improve the long-term durability of PLA biocomposites in real-world environments.

## 2. Materials and Methods

### 2.1. Materials

The formulation of the bio-based composites consisted of PLA, three different proportions of OBA (0%, 1%, or 3% wt.) and a process additive (1.5% wt.). Raw PLA (PLA_s_), Ingeo™ Biopolymer 3251D grade (NatureWorks LLC, Plymouth, MN, USA), confirmed to be ash-free (inorganic residue <0.1%) by TGA analysis, was used as a control and as the polymer matrix of the biocomposites. Drawing on prior findings reported by the authors [25], the weight proportions of 1% (PLA_b1_) and 3% (PLA_b3_) were selected, since these loadings achieved the optimal balance between mechanical reinforcement and processability. The Olive Pits (OPs), with commercial reference “Piropel Mix Biomasud A1”, used in the experiments were purchased from Pélaez Renovables (Jaén, Spain). According to the test report provided by CARTIF Technology Center (Valladolid, Spain), these OPs show the characteristics indicated in Table 1. Finally, the Coupling Agent (CA) BYK-P 4101 supplied by BYK-Chemie GmbH (Wesel, Germany) was employed to improve the injection molding processing and mechanical strength of the biocomposites.

### 2.2. Compounding and Processing

**Figure 1:**
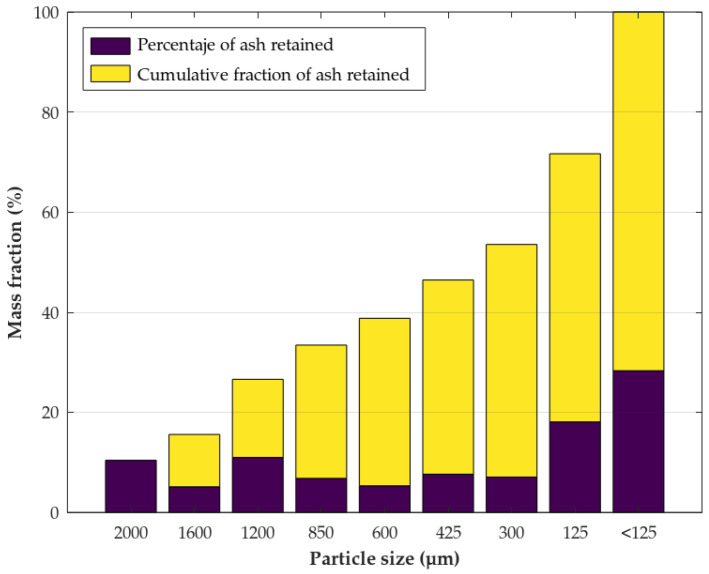
Granulometric distribution of ash particles obtained from OP combustion.

The ashes were generated by combusting OPs in a domestic boiler (Hidrocopper Super 29 kW, Ecoforest, Pontevedra, Spain), which typically operates at combustion temperatures in the range of 700–900 °C under standard atmospheric conditions. Subsequently, the solid residue consisting of the OPs resulting from combustion was subjected to a particle size separation process. A sieving system (BA200N, CISA Sieving Technologies, Barcelona, Spain) was used for this purpose, consisting of eight sieves with mesh apertures ranging from 2 mm to 0.125 mm (Figure 1). The fraction with a particle size below 0.125 mm, representing 28.33% of the total solid residue, was selected for the fabrication of the biocomposites investigated in this study. This finest fraction was chosen to promote a more homogeneous dispersion within the polymer matrix and to reduce the formation of large aggregates that could act as stress concentrators [26].

The ash and PLA were dried in order to remove moisture prior the compounding process. In the case of ashes, they were conditioned with a heater at 60 °C for 24 h. The PLA was dried in an industrial dehumidifier KKT 75 from KOCH-TECHNIK (Ispringen, Germany), which has two containers and a capacity of 75 m^3^/h dry air volume. The drying process conditions for PLA were selected according to the supplier’s instructions. Specifically, PLA granules were dried at 60 °C for 24 h.

The raw PLA pellets, the ash powder, and the additive were mixed using a Rehoscam Internal Mixer system (Scamex, Isques, France) with double nitrided stainless steel blade. A total mass of 150 g per batch was processed for 8 min at a screw speed of 60 rpm and 175 °C. Finally, the resulting homogeneous mass was ground with a WSGM-250 milling system (J. Puchades S.L., Albal, Valencia, Spain) equipped with a sieve of 8 mm mesh size.

**Figure 2:**
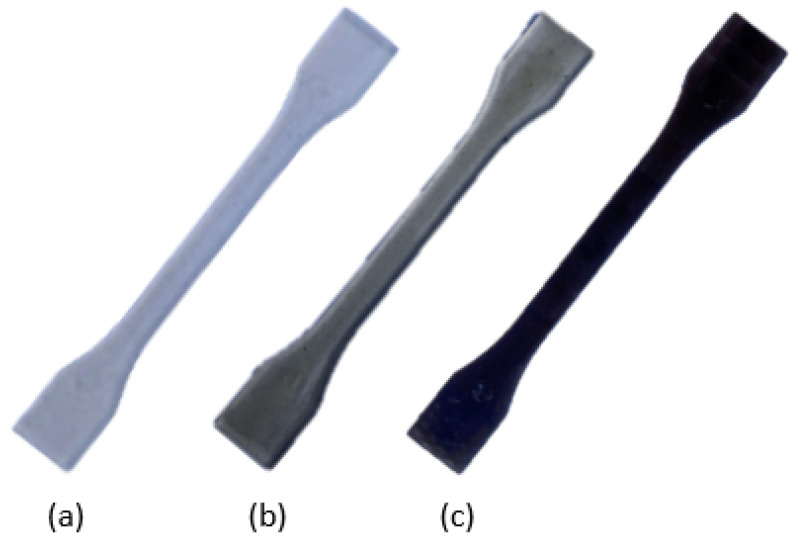
Tensile 1BA specimens of (**a**) PLA_s_, (**b**) PLA_b1_, and (**c**) PLA_b3_ manufactured by injection molding.

Before the molding process, the pellets of the resulting biocomposites were dried into the KKT 75 dryer in order to remove moisture. Then, using the ENGEL Victory 28 (Engel Holding GmbH, Schwertberg, Austria) injection molding machine, several specimens for each of the 3 material variants or batches (PLA_s_, PLA_b1_, and PLA_b3_; Figure 2) were manufactured according to ISO 527-2 (type 1BA) standard [27]. The injection molding parameters were set as follows: temperature profile 150 °C/155 °C/165 °C/nozzle 170 °C, mold temperature 70 °C, injection pressure 65 bar, screw speed 68 mm/s, holding time 10.5 s, and cooling time 25 s.

**Figure 3:**
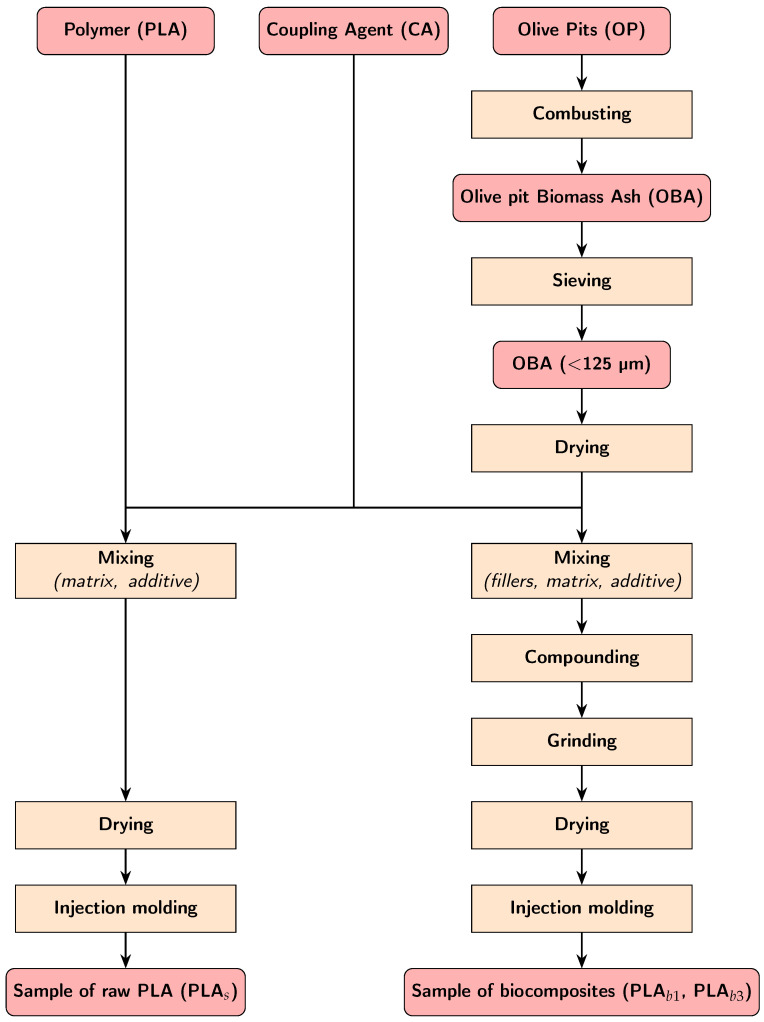
Flowchart illustrating the preparation process of PLA and OBA biocomposites. Pink boxes represent the materials and products, while beige boxes indicate the processing stages.

The preparation process for the PLA and OBA biocomposites followed the workflow illustrated in Figure 3.

### 2.3. Weathering Tests

Accelerated weathering tests were conducted in a xenon arc lamps (Q-SUN Xe-3-HSE) chamber from Q-lab (Westlake, OH, USA). Samples were subjected to an accelerated weathering cycle 1 for daylight filter following the specifications of ISO 4892-2 (Method A) standard [20]. Daylight-Q filter with a nominal cut-on of 295 nm was used, as it provides an accurate spectral match with direct sunlight and the best correlation between laboratory and natural outdoor exposures [28]. It should be noted that, even though the temperature of the Black Standard Thermometer (BST) was controlled at 65 ± 3 °C, the actual temperature of each sample was not directly measured, although it can indeed vary slightly depending on the material composition. Nevertheless, the BST provides a reproducible reference for the maximum surface temperature of the samples during exposure. The specimens were removed from the tester for physicochemical and appearance characterization after the following exposure times: 0, 500, and 1000 h.

### 2.4. Tensile Test

Prior to the measurements, the specimens were suitably conditioned for 24 h under the same conditions (23 ± 2 °C and 50 ± 10% RH). Tensile tests were performed in accordance with ISO 527-1 standard [29] using a Shimadzu (Kyoto, Japan) AGX-V universal testing machine equipped with a TRViewX non-contact digital video extensometer and a 5 kN load cell. A crosshead speed of 0.5mm/min was employed to allow precise monitoring of the modulus in the initial elastic region. At least five samples (type 1BA with 2.0 mm thickness) were tested for each type of material, and the results are presented as the average of the modulus of elasticity *E*, the yield strength $\Sigma_{y}$, and the yield strain $\varepsilon_{y}$.

#### 2.4.1. Effective Composite Moduli

The calculations of the Young’s modulus in particulate-filled polymer have been extensively analyzed in the literature [30,31]. Classical models as well as several empirical or semi-empirical equations have been developed to estimate their tensile modulus. One of the earliest theories for a composite system was an adaptation of Einstein’s spherical inclusions [32] given by Smallwood [33] for elastomers and applicable only to filled materials with loss concentrations of non-interactive spheres. Further, Guth [34] generalized the Einstein concept to account for inter-particle interactions at higher filler concentrations. The difficulties associated with the definition of these interactions lead to several modifications of the Einstein’s equation, with one of the best being the one proposed by Mooney [35], who, together with Frankle-Acrivos [36], derived the first equations to introduce the concept of maximum packing fraction to take into account differences in particle geometry. One of the most versatile and elaborate models for a composite material consisting of spherical particles in a matrix was derived by Kerner [37], which was modified by Halpin and Tsai [38,39,40] in order to find the modulus of the particle. And then, Nielsen [41] suggested a more general form in what is now known as the Halpin–Tsai–Nielsen equation:

(1) $$\frac{E_{b}}{E_{s}}=\frac{1+AB\phi_{v}}{1-B\psi \phi_{v}}$$

where the left-hand side corresponds to the relative elastic modulus (biocomposite modulus versus matrix modulus), $E_{s}$ is the tensile modulus of the matrix, ψ is dependent upon the particle packing fraction of the filler, while *A* and *B* are constant for any given composite. The constant *A* accounts for factors such as the shape of the dispersed particles, their state of agglomeration, their orientation, and the nature of the interface; *B* takes into account the relative modulus of the filler and the matrix phases.

(2) $$A=k_{E}-1$$

(3) $$B=\frac{E_{a}/E_{s}-1}{E_{a}/E_{s}+A}$$

(4) $$\psi =1+\left(\frac{1-\phi_{max}}{\phi_{max}^{2}}\right)\phi_{v}$$

with $k_{E}$ being the generalized Einstein coefficient, $E_{a}$ being the tensile modulus of the matrix, and $\psi \phi_{v}$ being a factor that enables one to use a reduced concentration scale to consider the existence of the maximum packing fraction $\phi_{max}$ of the particles.

However, if we review the Young’s modulus obtained from the experimental tensile test data, we can notice that the stiffness of the biocomposite decreases as the ash content increases. This indicates that the filler is not behaving as a rigid phase, thus being an inverted system, for which Nielsen [42] proposed a slight modification. For the inverted case, Equations (2)–(4) become the following:

(5) $$\frac{E_{s}}{E_{b}}=\frac{1+A_{i}B_{i}\phi_{v}}{1-B_{i}\psi \phi_{v}}$$

(6) $$A_{i}=\frac{1}{k_{E}-1}$$

(7) $$B_{i}=\frac{E_{s}/E_{a}-1}{E_{s}/E_{a}+A_{i}}$$

Substituting Equations (4)–(7) in Equation (5), we can write them as such:

(8) $$\frac{E_{s}}{E_{b}}-\frac{1+\left(\frac{1}{k_{E}-1}\right)\left(\frac{E_{s}/E_{a}-1}{E_{s}/E_{a}+\left(\frac{1}{k_{E}-1}\right)}\right)\phi_{v}}{1-\left(\frac{E_{s}/E_{a}-1}{E_{s}/E_{a}+\left(\frac{1}{k_{E}-1}\right)}\right)\left(\phi_{v}+\left(\frac{1-\phi_{max}}{\phi_{max}^{2}}\right)\phi_{v}^{2}\right)}=0$$

#### 2.4.2. Yield Strength

The strength of a material is defined as the maximum stress that the material can withstand under a uniaxial tensile load, which for the field of polymers usually coincides with the yield stress ($\sigma_{y}$). In the case of a porous matrix, these voids can weaken the material by creating stress concentrations, leading to nonlinear mechanical behavior and failure associated with plastic deformation, as well as void nucleation and growth processes. To this end, several works have been carried out in recent decades. Gurson [43] proposed a pioneering macroscopic criterion using a method of limit analysis in an ideal rigid-plastic von Mises-type matrix containing spherical or cylindrical voids. Based on this paper, a number of extensions were published, most notably, the modification of the Gurson’s analysis by Tvergaard–Needleman [44] and the generalization applied to spheroidal voids containing a confocal cavity [45], which introduced a better fit of the experimental evidence and numerical solutions. Most recently, new trial velocity fields inspired by the Eshelby equivalent inclusion problem [46] have been incorporated into ductile materials containing spheroidal voids [47], and the plastic anisotropy of the matrix has been taken into account [48,49].

As a fundamental difference to metallic materials, which usually show pressure-independent behavior, porous polymers can exhibit a pressure-dependent solid matrix due to internal friction. Some of the first heuristic macroscopic contributions formulated for porous materials taking into account the pressure sensitivity of the solid matrix were developed by Lazzeri–Bucknall [50] and Jeong–Pan [51]. Subsequently, due to its simple linear function, the Drucker–Prager criterion became the most widely adopted homogenization technique for predicting the macroscopic strength criteria of porous geo-materials and polymers, focusing on the modified secant approach [52] and the limit analysis method containing spherical [53] or spheroidal voids [54,55].

According to previous studies dealing with void shape effects [45,47], the unit cell can be defined as a single axisymmetric prolate ellipsoidal cavity or semi-major axis $a_{1}$ or as a minor semi-minor axes $b_{1}$ embedded in a medium having the shape of a confocal ellipsoid of semi-major axis $a_{2}$ and semi-minor axis $b_{2}$. The cavity shape is characterized by the aspect ratio γ ($a_{1}/b_{1}$) and its focal distance $c={\left(a_{1}^{2}-b_{1}^{2}\right)}^{1/2}$, which coincide with the focus of the external confocal spheroidal domain $c={\left(a_{2}^{2}-b_{2}^{2}\right)}^{1/2}$. On the other hand, starting from cylindrical coordinates associated with an elliptic orthonormal basis ($e_{\lambda }$, $e_{\varphi }$, $e_{\theta }$) allows us to obtain the major and minor semi-axes as a=c·cosh(λ) and b=c·sinh(λ) with λ∈[0, +∞], and where λ-iso surfaces define confocal spheroids with foci c and eccentricity e=c/a. The unit cell’s porosity (linked to the volume fraction) and the void shape ratio are expressed by

(9) $$\phi_{v}=\frac{V_{void}}{V_{unit\;cell}}=\frac{a_{1}b_{1}^{2}}{a_{2}b_{2}^{2}}=\frac{\text{cosh}(\lambda_{1})}{\text{cosh}(\lambda_{2})}{\left(\frac{\text{sinh}(\lambda_{1})}{\text{sinh}(\lambda_{2})}\right)}^{2}$$

(10) $$\gamma =\frac{a_{1}}{b_{1}}=\text{cosh}\left(\lambda_{1}\right)$$

Knowing $\phi_{v}$ and γ from the previous subsection, we can determine the eccentricities of the inner $e_{1}$ and outer $e_{2}$ ellipsoids. These values will help us to calculate the coefficients κ and μ derived from the fourth-order tensor T(λ), which is obtained from the Eshelby-like velocity fields and whose components are only functions of the λ coordinate. Using unified expressions for prolate cavities [47] gives us the following:

(11) $$\kappa =\frac{1-e_{i}^{2}}{e_{i}^{3}}{\text{tanh}}^{-1}\left(e_{i}\right)-\frac{1-e_{i}^{2}}{e_{i}^{2}},\;i=1,2$$

(12) $$\mu =\left(1-3\kappa \right)\frac{1}{e_{i}^{2}},\;i=1,2$$

Unlike von Mises-type materials, porous polymeric materials are pressure-sensitive, and their mechanical behavior depends not only on shear stress but also on hydrostatic pressure. In the context of limit analysis theory, such behavior can be described by a microscale yield criterion based on a linear combination of the mean stress $\sigma_{m}$ and equivalent stress $\sigma_{eq}$ as the Drucker–Prager type criterion, which is expressed mathematically as

(13) $$\Phi =\sqrt{J_{2}}+3\omega I_{1}-k\leq 0\to \Phi \left[\sigma \right]=\sigma_{eq}+3\omega \sigma_{m}-\sigma_{eff}\leq 0$$

where $\sigma_{m}=tr\left(\sigma \right)/3$ and $\sigma_{eq}={\left(\left(3/2\right)\sigma^{\prime }:\sigma^{\prime }\right)}^{1/2}$, with $\sigma^{\prime }$ being the deviatoric part of the local stress σ. The material constants ω and $\sigma_{eff}$ are the frictional coefficient and the effective strength due to the presence of cracks or damage in the matrix, respectively.

By considering an associated flow rule in the matrix, we shall get the following closed-form of the approximate macroscopic strength criterion for a porous medium with a Drucker–Prager-type matrix and spheroidal cavities [54] as follows:

(14) $$\Theta^{2}\left[\frac{\Sigma_{eq}^{2}}{\sigma_{eff}^{2}}+\frac{{\left(\tau \left(3\kappa_{2}-1\right)\frac{\Sigma_{eq}^{2}}{\sigma_{eff}^{2}}+\frac{\Sigma_{s}}{\Theta }+\frac{\Sigma_{q}}{\sigma_{eff}}\right)}^{2}+\left(\zeta -1\right)\frac{\Sigma_{q}^{2}}{\sigma_{eff}^{2}}}{1-\zeta }\right]+2\phi_{v}\;\text{cosh}\left(\frac{\Gamma }{p}\right)-1-\phi_{v}^{2}=0$$

with the expressions $\Sigma_{eq}$, $\Sigma_{s}$, and $\Sigma_{q}$ defined as a function of the macroscopic stress tensor Σ, which is related to the transverse isotropy invariants and can be calculated once the loading state is known. The effective yield strength $\sigma_{eff}$ is typically derived from specialized homogenization models in micromechanics or damage mechanics. And the other parameters involved are given according to Appendix B.

### 2.5. Color Measurements

The surface color of some tensile specimens for each batch was measured with a Color i7 spectrophotometer (X-Rite, Grand Rapids, MI, USA) according to ISO 11664 standard [56]. The equipment was set up with a D65 standard illuminant and 10° viewing angle (CIELAB color system). Lightness ($L^{*}$) and two chromatic coordinates ($a^{*}$ and $b^{*}$) were measured. The color difference ($\Delta E^{*}$) was calculated according to the following equation:

(15) $$\Delta E^{*}=\sqrt{{\left(\Delta L^{*}\right)}^{2}+{\left(\Delta a^{*}\right)}^{2}+{\left(\Delta b^{*}\right)}^{2}}$$

where $\Delta L^{*}$, $\Delta a^{*}$, and $\Delta b^{*}$ are the total changes, given by the differences between initial values ($L_{0}^{*}$, $a_{0}^{*}$, and $b_{0}^{*}$) and those obtained after subjecting the samples to accelerated weathering for a certain time ($L_{t}^{*}$, $a_{t}^{*}$, and $b_{0}^{*}$). An increase in the $L^{*}$ value means that the color of the sample becomes lighter, ranging from 100 (white) to 0 (black). A positive $\Delta a^{*}$ value means a color shift towards red, while a negative $\Delta a^{*}$ value means a color shift towards green. A positive $\Delta b^{*}$ value means a color shift towards yellow, and a negative $\Delta b^{*}$ value means a shift towards blue.

### 2.6. Surface Gloss Measurements

Surface gloss of the type 1BA samples was measured with a PCE-SGM 60-ICA glossmeter (PCE Instruments, Meschede, Germany). The test angle was 60° according to ISO 2813 [57], and the measured results were expressed in gloss units (GUs). Gloss was measured at five different locations for one sample from each batch.

### 2.7. ATR-FTIR

Molecular structures on the surface of the samples were monitored by a Fourier transform infrared (FT-IR) spectrometer (Bruker Tensor 27, Ettlingen, Germany) equipped with a diamond attenuated total reflection (ATR) cell and a KBr beam splitter. For each sample, 32 scans were recorded in absorbance units with a resolution of $4\;{\mathrm{cm}}^{-1}$ from 4000 to $600\;{\mathrm{cm}}^{-1}$ and collected at a minimum of three different locations. Spectra were averaging after baseline correction and smoothing (5 points) of the entire spectrum with OPUS 8.7.31 software. Analysis of the carbonyl and hydroxyl bands is considered as a measure of the degree of weathering, as it is well established that both grow upon photodegradation. To quantify the Carbonyl Index (CI), absorption spectra of carbonyl (C=O) band area (1850–$1650\;{\mathrm{cm}}^{-1}$) were analyzed and normalized to a methylene (CH_2_) reference band area (1500–$1420\;{\mathrm{cm}}^{-1}$) associated with C–H deformation [58]. Similarly, the Hydroxyl Index (HI) was calculated using the hydroxyl (OH) absorption band area (3700–$3230\;{\mathrm{cm}}^{-1}$) and the scissoring reference band area [59].

(16) $$CI=\frac{\mathrm{Area}\;\mathrm{under}\;\mathrm{band}\;1850–1650\;{\mathrm{cm}}^{-1}}{\mathrm{Area}\;\mathrm{under}\;\mathrm{band}\;1500–1420\;{\mathrm{cm}}^{-1}}$$

(17) $$HI=\frac{\mathrm{Area}\;\mathrm{under}\;\mathrm{band}\;3700–3230\;{\mathrm{cm}}^{-1}}{\mathrm{Area}\;\mathrm{under}\;\mathrm{band}\;1500–1420\;{\mathrm{cm}}^{-1}}$$

### 2.8. Scanning Electron Microscopy (SEM)

Surface morphology of the samples during weathering was monitored by a Carl Zeiss Merlin (Jena, Germany) high-resolution field-emission scanning electron microscope (FE-SEM) with an acceleration voltage of 2 kV. Prior to analysis, the specimens were cut into pieces of approximately 5 mm × 5 mm, and their weathered surfaces were sputter-coated with gold.

### 2.9. Thermal Analysis

Thermal Gravimetric Analyzer (TGA) with Differential Scanning Calorimetry (DSC) experiments were performed using an SDT Q600 V20.9 Build 20 apparatus supplied by TA Instruments (New Castle, DE, USA). Each sample of approximately 15–30 mg was heated and cooled at a heating rate of 10 °C per minute under nitrogen (N_2_) atmosphere (20 mL/min) according to the following procedure: (a) heated from 30 °C to 200 °C, (b) held at 200 °C for 10 min, (c) cooled from 200 °C to 20 °C, and finally (d) warmed up to 700 °C.

(18) $$\chi_{c}=\frac{\Delta H_{m}-\Delta H_{cc}}{\Delta H_{m}^{0}\left(1-\varphi \right)}\;\cdot \;100$$

where $\Delta H_{m}^{0}=93.7\;J/g$ for a 100% crystalline PLA [60], $\Delta H_{m}$ is the melting enthalpy of the material, $\Delta H_{cc}$ is the cold crystallization enthalpy of the material, and φ expresses the weight fraction of ash in the biocomposite.

### 2.10. Kinetics

The majority of kinetic methods used in the solid thermal analysis consider the reaction rate (dα/dt) to be a function of only two process variables: temperature and reacted conversion.

(19) $$\frac{d\alpha }{dt}=K\left[T\right]f\left[\alpha \right]$$

where α is the decomposed fraction of the solid at time *t*; f[α] is a function of α, depending on the reaction mechanism; and K[T] is the temperature dependence of the process rate. The latter term is typically parameterized through the Arrhenius equation as follows:

(20) $$K\left[T\right]=Ae^{\frac{-E_{a}}{RT}}$$

with *A* being the pre-exponential factor (associated with the frequency of vibrations), *R* being the universal gas constant, and $E_{a}$ being the activation energy (related to the energy barrier). For constant heating rate (β) non-isothermal conditions, Equation (19) is often rearranged as

(21) $$\frac{d\alpha /dT}{f[\alpha ]}=\frac{A}{\beta }e^{\frac{-E_{a}}{RT}}$$

Integrating of Equation (21) leads to

(22) $$g\left[\alpha \right]\equiv \int_{0}^{\alpha }\frac{d\alpha }{f[\alpha ]}=\frac{A}{\beta }\int_{T_{0}}^{T}e^{\frac{-E_{a}}{RT}}dT$$

where g[α] is the integral form of the reaction mode. It is important to note that the right-hand side is limited to processes where the sample temperature does not deviate significantly from the reference temperature. Because the temperature term is generally considered not to be analytically integrable, a number of approximate solutions were offered in the past [61,62,63]. In the following, we will make use of the exponential integral function ($E_{i}$), which allows one to avoid its explicit evaluation.

Make the substitution $\Lambda =E_{a}/RT$ to obtain $d\Lambda =-E_{a}/RT^{2}$ [64]. Therefore, the right-hand side of Equation (22) becomes

(23) $$\frac{A}{\beta }\int_{T_{0}}^{T}e^{\frac{-E_{a}}{RT}}dT=-\frac{A}{\beta }\frac{E_{a}}{R}\int_{\Lambda_{0}}^{\Lambda }\frac{e^{-\Lambda }}{\Lambda^{2}}d\Lambda =\frac{A}{\beta }\frac{E_{a}}{R}\left(\frac{e^{-\Lambda }}{\Lambda }-\frac{e^{-\Lambda_{0}}}{\Lambda_{0}}-E_{i}\left[\Lambda \right]+E_{i}\left[\Lambda_{0}\right]\right)$$

Undoing the change of variable and substituting in Equation (22) results in

(24) $$g\left[\alpha \right]=\frac{A}{\beta }\left(Te^{\frac{-E_{a}}{RT}}-T_{0}e^{\frac{-E_{a}}{RT_{0}}}-\frac{E_{a}}{R}\left(E_{i}\left[\frac{E_{a}}{RT}\right]-E_{i}\left[\frac{E_{a}}{RT_{0}}\right]\right)\right)$$

The next step will be to fit this Equation (24) to the sigmoidal experimental curve (α vs. *T*) in order to obtain the kinetic triplets (*A*, $E_{a}$, and mechanism), an objective which different reaction models have historically. However, we will focus on the Avrami–Erofeev models, as this is the recommended method [65] capable of reproducing more reliably autocatalytic behavior, i.e., reaction profiles whose initial and final stages show an acceleration and a deceleration, respectively.

Fitting has been carried out by means of a nonlinear regression that minimizes the difference between the calculated and experimental values. The least-squares solver evaluates the difference in the form of the Residual Sum of Squares (RSS), or in other words, the squared Euclidean norm:

(25) $$RSS=\min_{E_{a},A}{∥\alpha_{calc}\left[E_{a},A,T\right]-\alpha_{exp}\left[T\right]∥}_{2}^{2}=\min_{E_{a},A}\sum_{i}{\left(\alpha_{calc}\left[E_{a},A,T_{i}\right]-\alpha_{exp}\left[T_{i}\right]\right)}^{2}$$

where $\alpha_{calc}$ refers to the conversion calculated by Equation (24), and $\alpha_{exp}$ refers to those values obtained from the experimental TGA data. Then, a minimum residual is numerically found by varying the kinetic parameters from an initial estimate of $E_{a}$ and *A* values. Additionally, to compare the goodness of fit between various reaction mechanisms f[α], the variance can be used, $S^{2}$, as

(26) $$S^{2}=\frac{RSS}{n-p}$$

with *n* being the number of points considered in the calculation and *p* being the set of kinetic parameters to be considered.

### 2.11. Acceleration Factor (AF)

A recurring question is the following: how much outdoor time is the laboratory test equivalent to? It is best to consider how an accelerated test corresponds to a standard outdoor exposure site (see Table 2) such as Miami (Florida) or Phoenix (Arizona) and then estimate how Miami or Phoenix relates to actual use in other locations [21].

The acceleration factor for photodegradation ($AF_{light}$) is proportional to the ratio between the total effective UV dosage that the exposed material received in the test chamber ($D_{acc}$) and the actual outdoor conditions ($D_{out}$) during the same period of time *t* [22,23]:

(27) $$AF_{light}={\left(\frac{D_{acc}\left[t\right]}{D_{out}\left[t\right]}\right)}^{p}$$

where *p* is the Schwarzschild coefficient, which considers the nonlinearity of material response to intensity. Intuitively, this total effective dosage can be thought of as the cumulative number of photons absorbed into the degrading sample and that cause chemical change:

(28) $$D\left[t\right]=\int_{0}^{t}\int_{\lambda_{1}}^{\lambda_{2}}d\left[\lambda ,\tau \right]d\lambda d\tau$$

with d[λ,τ] representing the instantaneous effective dosage of photons with wavelength λ at real time τ, which is usually calculated as the spectral irradiance or intensity, I[λ,τ], and typically taken over the UV band (295 nm to 385 nm). This approach is simple and good for, as in our case, the chamber’s arc lamp has a UV spectrum that matches well with natural sunlight [66]. Then, the total effective UV dosage, considering that both artificial and natural light sources have time-independent mixtures of light at different wavelengths, is given by

(29) $$D\left[t\right]=\int_{0}^{t}\int_{\lambda_{1}}^{\lambda_{2}}I\left[\lambda ,\tau \right]d\lambda d\tau =t\int_{\lambda_{1}}^{\lambda_{2}}I\left[\lambda \right]d\lambda =Et=H$$

with *E* being the spectrum energy between 295 and 385 nm and *t* being the exposure time.

On the other hand, photothermal degradation generally proceeds faster as the temperature increases [67,68], following the Arrhenius equation, the acceleration factor can be expressed as the ratio of the rates as follows:

(30) $$AF_{temp}=\frac{k_{acc}}{k_{out}}=\frac{e^{\frac{-E_{a}}{RT_{acc}}}}{e^{\frac{-E_{a}}{RT_{out}}}}=e^{\frac{E_{a}}{R}\left(\frac{1}{T_{out}}-\frac{1}{T_{acc}}\right)}$$

where $E_{a}$ is the activation energy of the photothermal reaction calculated in the previous subsection by experimental data fitting, *R* is the gas constant; $T_{acc}$ and $T_{out}$ define the effective temperature at the exposed surface of the sample for the test chamber and outdoors, respectively.

Finally, in order to take into account the effect of the humidity on the sample, the Peck’s relationship [69] can be considered as follows:

(31) $$AF_{hum}={\left(\frac{RH_{acc}}{RH_{out}}\right)}^{n}$$

where $RH_{acc}$ and $RH_{out}$ represent the humidity levels, and *n* reflects the sensitivity of the degradation rate. The overall acceleration due to intensity increase, the rise in temperature, and the humidity impact can be written as

(32) $$AF_{total}=AF_{light}\times AF_{temp}\times AF_{hum}={\left(\frac{H_{acc}}{H_{out}}\right)}^{p}e^{\frac{E_{a}}{R}\left(\frac{1}{T_{out}}-\frac{1}{T_{acc}}\right)}{\left(\frac{RH_{acc}}{RH_{out}}\right)}^{n}$$

The exposure time is then predicted for the exterior from the equivalent accelerated weathering test duration as follows:

(33) $$t_{out}=t_{acc}\times AF_{total}$$

## 3. Results and Discussion

### 3.1. Weathering Tests

PLA, an aliphatic polyester, is composed of repeating units of lactic acid linked by ester bonds [70]. Its chemical structure features characteristic functional groups, including ester linkages (–COO–), carbonyl groups (C=O), and methyl groups (–CH_3_) [71]. This aliphatic polyester is known for its biodegradability, making it a sustainable alternative to conventional plastics. However, its ester groups are susceptible to hydrolysis and photolysis, resulting in faster degradation under outdoor conditions compared to traditional polymers. Accelerated weathering tests have been conducted to evaluate the behavior of PLA and its biocomposites over time, and it has been concluded that aging is caused by a combination of photodegradation processes, leading to significant changes in their physical, thermal, and mechanical properties [3,12,13].

#### 3.1.1. Photolysis

The research [13,72] consistently identifies Norrish II photolysis as the predominant mechanism for PLA photodegradation. This absorption triggers a photochemical reaction where a photon excites the polymer, causing the scission of the C–O bond in the ester groups. This intramolecular process forms carboxylic acid and diketone end groups, initiating random chain scission mainly in the amorphous regions of PLA, where polymer chains are less ordered and more accessible to UV light compared to crystalline regions. In addition, photolysis of diketones promotes the hemolytic cleavage of the C–C bond between the two carbonyl groups. The Norrish II mechanism involves the formation of radicals that propagate to the ester bond, which further aggravates the degradation. This is especially significant, because it leads to a drastic reduction in molecular weight, weakening the mechanical properties of the matrix, such as tensile strength and ductility, which facilitates the formation of surface microcracks. On the other hand, natural fillers may alter photodegradation behavior of the material, functioning either as UV absorbers or scattering centers that partially protect the matrix [73], or alternatively by accelerating degradation due to poor interfacial adhesion and stress concentration at the filler/matrix interface [74].

#### 3.1.2. Hydrolysis and Thermal Effects

The inclusion of moisture cycles, simulating rain and dew, involves the cleavage of ester bonds, occurring predominantly in the amorphous regions due to their greater permeability and accessibility to water molecules. This autocatalyzed process is intensified by the presence of carboxylic acid end groups, which serve as reactive sites for hydrolytic attack. Degradation occurs both at the exposed surfaces, due to high water accessibility, and within the amorphous domains of the polymer bulk, where it is promoted by the accumulation of acidic by-products [11,12]. Studies [13,72] indicate that this mechanism further reduces molecular weight, compromising mechanical integrity. Additionally, the biocomposite filler absorbs moisture, which causes it to swell. This swelling exerts mechanical stress on the surrounding PLA matrix, causing cracks to form and propagate [17].

Temperature plays a key role in the rate and extent of hydrolysis [72]. At 35 °C, degradation is observed to predominantly affect the amorphous regions, with a gradual increase in crystallinity and glass transition temperature. At 45 °C, degradation initially targets the amorphous parts but subsequently affects the crystalline regions, showing both growth and decay phases for crystallinity and transition temperature. This biphasic behavior suggests a complex interaction between temperature and PLA’s structural phases. Above its glass transition temperature (around 60–65 °C), molecular mobility in PLA increases. At higher temperatures, typically above 100 °C, PLA is also subject to thermo-oxidative degradation mechanisms, where the cleavage occurs at the tertiary C–H bond, which presents the lowest dissociation energy [75].

#### 3.1.3. Cyclic Effects

Weathering is not a static process; it involves cyclic variations in temperature and humidity that impose fatigue stresses on the exposed surface. These cyclic changes can create thermal and moisture gradients, leading to non-uniform dimensional changes and internal stresses. Such stress fatigue, combined with molecular weight reduction due to photodegradation, might lower the fatigue limit at microsites, initiating and propagating microcracks [76]. In addition to warping and loss of dimensional stability, especially in biocomposites where thermal expansion differences between the matrix and inhomogeneities are relevant—as well as poor filler/matrix interface—adhesion [77] can exacerbate damage [78].

The weathering of PLA results in a variety of degradation products, reflecting the different chemical reactions that take place [79]. One of the primary products is lactic acid, the monomer from which PLA is made, which is formed through the hydrolytic cleavage of the polymer chains. Along with lactic acid, oligomers, which are shorter chains of lactic acid units, are also produced during hydrolysis and chain scission. Under complete degradation, PLA can be broken down into its ultimate constituents of carbon dioxide CO_2_ and water H_2_O. Photo-oxidation and thermal degradation lead to the formation of various aldehydes, ketones, and carboxylic acids, as oxygen is incorporated into the polymer fragments. Finally, the physical stresses and chemical breakdown caused by weathering can lead to the fragmentation of the PLA material into smaller pieces, resulting in the formation of microplastics [80].

Then, when factors like UV radiation, temperature, and humidity are combined, hydrolysis and photolysis are exacerbated, leading to noticeable surface defects and a significant reduction in molecular weight. Exposure to UV radiation of wavelengths of 400 nm or lower can cleave C–O and C–C covalent bonds in PLA, generating radicals and inducing chain scission that markedly decrease molecular weight. While radical recombination or limited branching may occur, available evidence indicates that chain scission is the primary degradation mechanism under weathering conditions [59]. This prevailing pathway frequently leads to physical alterations (color and gloss), embrittlement, microcracking, and reduced mechanical properties. The aging behavior of unfilled PLA is typically simpler than that of PLA-based biocomposites, where components like fillers, fibers, additives, plasticizers, and antioxidants complicate degradation mechanisms [81]. The following sections will discuss the mechanical, chemical, and physical performance of these materials.

### 3.2. Tensile Test

#### 3.2.1. Effective Composite Moduli

Young’s modulus is a measure of stiffness that is crucial to understanding how PLA and its biocomposites behave under weathering conditions, such as UV light cycles, humidity, and temperature variations. These factors can cause photodegradation and hydrolysis, altering PLA’s molecular structure and mechanical properties. Studies suggest that the effect of weathering on moduli is not uniform and depends on the type of measurement (bulk or surface) and the duration of exposure. Key research found that the mechanical properties of raw PLA were significantly reduced after 400 h of exposure, implying a likely decrease in the tensile modulus due to chain scission, which reduced the molecular weight and weakened the stiffness of the material [82]. In contrast, another study provided detailed surface Young’s modulus data by atomic force microscopy (AFM), indicating for pure PLA an increase in surface modulus with exposure time attributed to an increase in crystallinity due to chain rearrangement during degradation, making the surface stiffer but more brittle [83].

Knowing the volume per tensile specimen ($V=1.0388{\mathrm{cm}}^{3}\;)$ and the averaged dry mass (m) from Table 3 of the unweathered PLA_s_ control samples, we can calculate the density for the raw PLA as ρ=m/V, which turns out to be $\rho_{s}\left[0\;h\right]=1.2403\;g/{\mathrm{cm}}^{3}\;,$ corresponding to the value provided by the supplier’s data sheet. Similarly, we can determine the densities of the biocomposites as $\rho_{b1}\left[0\;h\right]=1.2373$ $g/{\mathrm{cm}}^{3}$ and $\rho_{b3}\left[0\;h\right]=1.2096\;g/{\mathrm{cm}}^{3}$. Then, the bulk density of filler or OBA can be obtained according to the linear rule of mixtures [84] as follows:

(34) $$\rho_{b}=\frac{\rho_{s}\rho_{a}}{\rho_{s}m_{a}+\rho_{a}\left(1-m_{a}\right)}\to \rho_{a}=\frac{\rho_{b}\rho_{s}\phi_{m}}{\rho_{s}-\rho_{b}\left(1-\phi_{m}\right)}$$

where $\rho_{b}$, $\rho_{a}$, and $\rho_{s}$ are the densities of the biocomposite, filler, and matrix, respectively, and $\phi_{m}$ is the mass fraction of OBA. Thus, we would have that the ash density values for each OBA composition come out to $\rho_{a1}\left[0\;h\right]=0.9983\;g/{\mathrm{cm}}^{3}$ and $\rho_{a3}\left[0\;h\right]=0.6719\;g/{\mathrm{cm}}^{3}\;\;$ The difference between the two values can be explained, for example, by air inclusions or that the filler influences the density of the polymer phase by nucleation of crystal growth. And finally, the volume fraction of the filler can be computed by means of the expression $\phi_{v}=\left(\rho_{s}\phi_{m}\right)/\phi_{a}$, resulting in $\phi_{v1}=1.24\%$ for PLA_b1_ and $\phi_{v3}=5.54\%$ for PLA_b3_.

**Figure 4:**
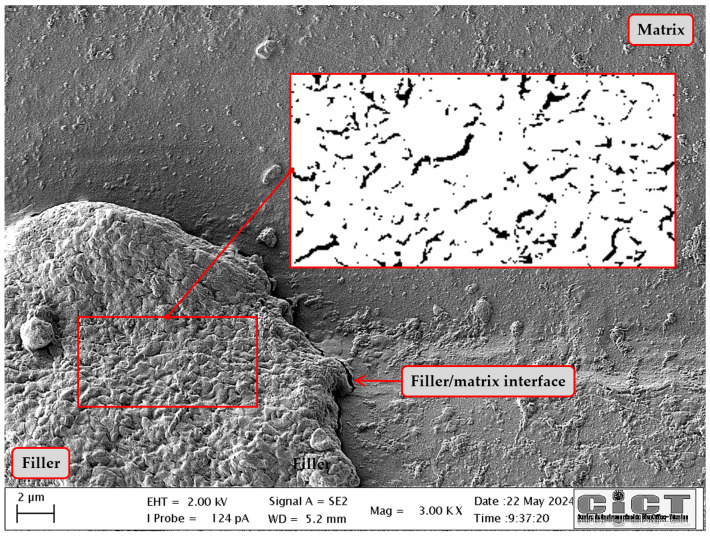
SEM image of the unexposed surface of a PLA_b1_ sample. An amplification obtained with ImageJ 1.54g [88,89] on the filler surface, which shows in white the particles area and in black the voids region.

**Figure 5:**
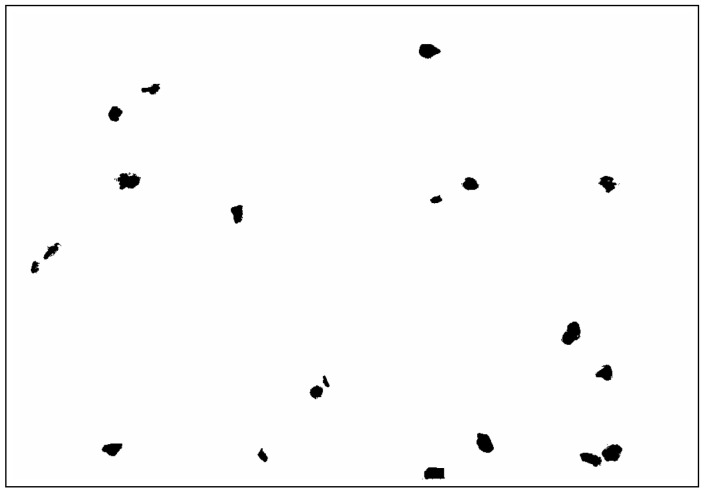
Image processed with a trainable weka segmentation algorithm [89,90] for the unexposed surface of a PLA_b1_ sample; Mag. 100×. Fillers (black color) on the matrix (white background).

A priori, three variables ($E_{a}$, $k_{E}$, and $\phi_{max}$) in Equation (8) are unknown. Although by examining the SEM image (Figure 4), we can point out that the filler shows an irregular and not very compact structure or voids typical of a particle agglomeration. From there, it can be deduced that the maximum packing fraction is really close to the 0.37 value [42]. In the case of the Einstein coefficient, it is more complicated to reach a theoretical value, as it depends on several factors such as (a) the specific geometry of the agglomerates, (b) the arrangement of primary particles within the agglomerate, and (c) the overall aspect ratio of the agglomerate. Some papers [85,86,87] showing good agreement between theory and experiment consider the geometry of the particles and their orientation with respect to the flow direction as relevant factors. Thus, treating each cluster as a particle with given orientation and shape determined by its Ferer’s AR (ratio of the maximum to the minimum Feret diameter), we could deduce from Figure 5 that in our case the filler is composed of agglomerations with random arrangement and a mean Ferer’s AR∼1.7. Therefore, an average Einstein coefficient for the OBA filler can be obtained as a value equivalent to the randomly oriented rods for AR=1.7, resulting in $k_{E}=2.6$ [42].

Once the experimental moduli of the matrix $E_{s}$ and the biocomposite $E_{b}$ are known (see Table 4), and the volume fraction $\phi_{v}$, the maximum packing fraction $\phi_{max}$, and the generalized Einstein coefficient $k_{E}$ are estimated, we could calculate the filler modulus $E_{a}$ from the inverted system Nielsen’s formula. Since this is a nonlinear equation, we have used one of Octave’s simplest minimizers [91]; more specifically, we have worked with the solver “fminbnd”, which allows us to find the minimum value of a univariate function for a knows search interval. Solving Equation (8) shows that the resulting value of $E_{a}$ tends to zero for both biocomposites; this makes us realize that the filler is behaving like a filler itself, but without providing adequate adhesion of the polymer to the filler surface, as well as weak cohesive forces between agglomerated particles. This means that macroscopically speaking, we can state that the ash-loaded samples are showing a mechanical behavior characteristic of a solid with a dispersion of soft inclusions or voids [92,93]. This allows the constitutive modeling of these composites to be greatly simplified by reducing heterogeneous structures to a single mean field or effective medium theory (EMT). The EMT supposes that the composite system is a homogeneous medium that has the same properties or physical constants [94,95], including its elastic modulus.

Several micro-mechanical models have been proposed for predicting the effective macroscopic properties for polymers with voids or inclusions. Historically, two key methods within EMT framework are Eshelby’s theory and the differential effective medium (DEM) approach. On the one hand, Eshelby’s inclusion solves the elasticity problem for an ellipsoidal inclusion in an infinite matrix [46,96]; on the other hand, the DEM incrementally adds inclusions to a medium, allowing for the calculation of effective properties as the concentration of inhomogeneities increases [97,98]. That is, while the first theory is ideal for understanding the impact of inclusions in the overall material, the second one is suited for modeling incremental changes in material properties, which makes them generally appropriate for low and high concentrations, respectively.

Eshelby’s theory underpins many later models, such as the self-consistent (SC), the generalized self-consistent (GSC), and the Mori–Tanaka (MT) model. The latter is one of the most prominent extensions of Eshelby’s solution [99]. The key idea behind the MT scheme is to calculate the average internal stress in the matrix by transforming the strain, accounting for interactions among inclusions and the effect of a free boundary on the average elastic energy [100], which means that if a porous body (filler elastic constants, $E_{a}=G_{a}=K_{a}\to 0$) is subjected to a uniform far-field stress $\sigma^{\infty }$ on its outer boundary, the mean stress in the matrix phase will be $\sigma^{\infty }/\left(1-\phi_{v}\right)$ [101]. Assuming an isotropic matrix with randomly oriented prolate spheroidal (b=c, y=a/b>1) pores, the usual energy considerations that are invoked in effective medium calculations [102] then lead to the following expressions for the effective bulk ($K_{eff}$), shear ($G_{eff}$) and Young’s moduli ($E_{eff}$):

(35) $$K_{eff}=\frac{K_{s}}{1+\frac{\phi_{v}}{1-\phi_{v}}P}$$

(36) $$G_{eff}=\frac{G_{s}}{1+\frac{\phi_{v}}{1-\phi_{v}}Q}$$

(37) $$E_{eff}=\frac{9K_{eff}Geff}{3K_{eff}+G_{eff}}$$

with $K_{s}$ and $G_{s}$ being the shear and bulk moduli of the matrix, respectively, which for isotropic materials are related with to the matrix Young’s modulus by $E_{s}=3K_{s}\left(1-2\nu_{s}\right)=2G_{s}\left(1+\nu_{s}\right)$, where the Poisson’s ratio ($\nu_{s}$) value for Ingeo™ Biopolymer 3251D is given by the constant 0.36 according to NatureWorks Co. (Plymouth, MN, USA) technical data sheet database [1]. For the elastic compliance of spheroidal pores, the compressibility *P* and the shear *Q* are cumbersome combinations of Eshelby–Wu tensor [103] components that can be rearranged as closed-form solutions dependent on the averaged aspect ratio γ and the Poisson ratio of the matrix material $\nu_{s}$, which are given in Appendix A.

Due to smallness OBA concentration ($\phi_{v}<<1$), the dilute limit condition can be assumed, as well as negligible interaction between the fillers or non-interaction approximation (NIA). In this case, Equations (35) and (36) adopt a slight simplification by complying with the ***H***-tensor formalism [104]:

(38) $$K_{eff}\approx \frac{K_{s}}{1+\phi_{v}P}$$

(39) $$G_{eff}\approx \frac{G_{s}}{1+\phi_{v}Q}$$

Next, based on the experimental data of Young’s modulus (see Table 4), the average aspect ratio is estimated from Equation (37) for unexposed samples, resulting in a typical value of γ=1.74, which, if we recall the AR calculated from Figure 5, is a value quite close to the same value. The discrepancy of the latter equivalent value with respect to the AR previously calculated from the SEM image is explained by the intrinsic limitations associated with the information that can be extracted from a 2D magnification, where the study region is restricted to a specific area and surface of the solid. In contrast, the analytical homogenization solution is endowed with a bulk property and set as an effective mean to the complete domain. Therefore, γ can be considered a more representative arithmetic value of the aspect ratio for the prolate spheroidal inhomogeneities present in these biocomposites.

In addition, the diluted MT scheme will allow us to assess the effective elastic moduli of the aged samples, but for this, it will be necessary to have previously known the type of defect that mostly takes place during the accelerated weathering test. According to the literature [82,105], when a PLA matrix is subjected to weathering, the most common finding is the degradation of the amorphous regions, driven by photolytic, hydrolytic, and thermo-oxidative mechanisms, leading to the origin of microcracks on the exposed surface due to chain scission that can propagate into larger fractures (crazing), which are known as slit-like cracks. Randomly distributed slit-like cracks are often modeled as a long narrow elliptic crack (b/a→0) of the effective Young’s modulus [106] as follows:

(40) $$\bar{E}=\frac{E_{eff}}{1+\frac{16}{45}\left(1+\nu_{s}\right)\left(5-4\nu_{s}\right)}$$

In contrast to the volume fraction of spheroidal pores, cracks typically have negligible volume; thus, their evaluation yields the crack density $\epsilon =\left(\pi /2\right)N\langle ab^{2}\rangle$ (*N* is the number of cracks per unit volume, and the angle brackets denote an average). Then, Equation (40) represents the extended effective elastic modulus ($\bar{E}$) of solids containing two populations of pores, spheroidal voids (fillers), and flat cracks (weathering defects) under the assumption of dilute concentrations (low porosity and crack density). In this case, we only want to evaluate the behavior of raw PLA (without OBA content) during weathering, the extended effective modulus ratio $\bar{E}/E_{eff}$ will be simplified to $\bar{E}/E_{s}$ (with the constant $E_{s}$ referring to the Young’s modulus of the PLA matrix prior to exposure).

It is then established that, for biocomposites, the volume fraction remains constant during the accelerated weathering test, which is a quite reasonable assumption for a homogeneous distribution of the biocomposite, since as material is shed or removed from the specimen, it should be in the same proportion of both the matrix and filler. This last consideration will allow us, using Equations (37) and (40) as well as the experimental data in the third column of Table 4, to estimate the crack density that occurs during the exposure of the different material variants, as indicated in Table 5.

Based on Table 5, the trend in density crack when the samples are subjected to weathering is an increase, which is driven by degradation processes that create more defects and weaken the structural integrity. While changes in crystallinity may reduce porosity in some cases, the dominant effect appears to be the formation of additional voids due to molecular decomposition driven by the processes of hydrolysis, photolysis, thermo-mechanical heating/cooling cycles, and rain erosion. In turn, we can state that the incorporation of fillers facilitates slit-crack initiation and propagation due to a poor filler/matrix interface, resulting in a higher density.

#### 3.2.2. Yield Strength

For any polymer matrix with a known yield strength $\sigma_{0}$ that undergoes weathering, cracks or micro-defects are expected to occur, which will introduce stress concentrations that will reduce the yield strength compared to an unweathered or undamaged material, leading when considering the dilute limit (where cracks are sparse and interactions are negligible) to the following linear approximation [107]:

(41) $$\sigma_{eff}=\sigma_{0}\left(1-D\right)\to \sigma_{eff}=\sigma_{0}\left(1-\varsigma \epsilon \right)$$

where *D* is the damage parameter, linked to the total crack surface area per unit volume, which can be calculated from the crack density ϵ and the dimensionless constant ς. The constant depends on (a) crack geometry, which involves long narrow elliptic or slit-like cracks that have high stress concentrations at their tips due to their aspect ratio and (b) material properties, which are influenced by the elastic modulus and Poisson’s ratio, as these affect how stress is distributed around the cracks. The value of ς can be estimated from the raw PLA experimental data (Table 4), making $\sigma_{0}$ the yield stress of undamaged samples ($\Sigma_{y,s}\left[0\;h\right]$) and $\sigma_{eff}$ the strength of weathered specimens ($\Sigma_{y,s}\left[500\;h\right]$ and $\Sigma_{y,s}\left[1000\;h\right]$), resulting in an average between the two time points of ς=15.52.

As far as composites are concerned, the macroscopic yield criterion for a Drucker–Prager matrix with randomly oriented spheroidal voids is too complex to be derived directly by integrating Equation (14) over the surface, but it can nevertheless be approximated using the criterion for spherical voids, since the random orientation may average out shape effects to make the material behave isotropically at the macroscopic level [54], giving the following equation:

(42) $$\frac{\frac{\Sigma_{eq}^{2}}{\sigma_{eff}^{2}}}{{\left(1-\frac{3\omega }{\left(1-\phi_{v}\right)}\frac{\Sigma_{m}}{\sigma_{eff}}\right)}^{2}}+2\phi_{v}\text{cosh}\left(\frac{2\omega +sgn(\Sigma_{m})}{2\omega }\ln\left(1-3\omega \frac{\Sigma_{m}}{\sigma_{eff}}\right)\right)-1-\phi_{v}^{2}=0$$

This equation makes it feasible to calculate the pressure sensitivity or friction angle ($\tan(\psi_{\omega })=3\omega$) from the experimental yield data and the estimated theoretical values for the volume fraction (see Table 6), taking into account that for a uniaxial tensile test the equivalent and mean macroscopic stress are simplified to $\Sigma_{eq}=\Sigma_{y}$ and $\Sigma_{m}=\Sigma_{y}/3$, respectively.

A slight downward trend in internal friction is observed as the exposure time increases, with the decrease being somewhat more pronounced for the biocomposite with higher filler content. A logical explanation for this angle decay over time is the degradation of the filler/matrix interface and loss of interlocking. Some effects of weathering could be the following: (a) the loss of cohesion, hydrolysis, and chain scission that reduce polymer entanglement, weakening the matrix binding fillers; (b) pore wall degradation that causes thinning of the interface that reduces interlocking; (c) microcrack propagation that causes stress concentration at the pores which accelerates crack growth, diminishing shear strength.

**Figure 6:**
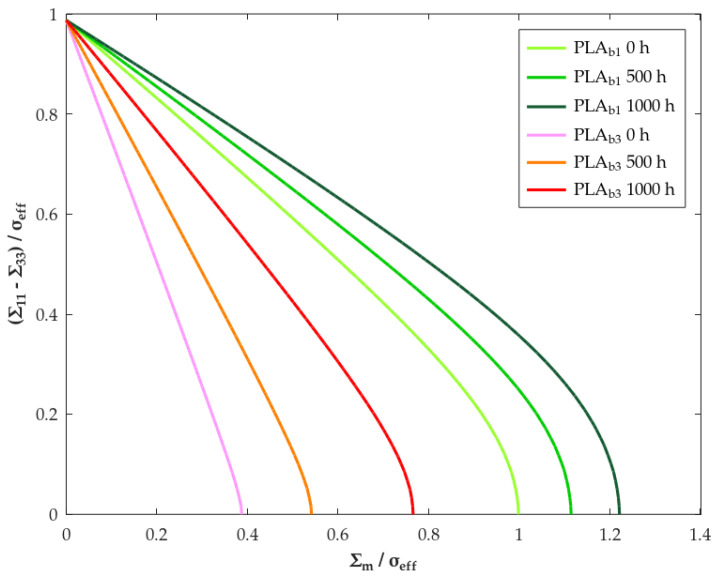
Yield surfaces predicted by Equation (14) for a prolate spheroidal void embedded in a matrix of yield strength $\sigma_{eff}$, with axisymmetric loading and compressibility given by Table 6.

The yield surfaces predicted by the macroscopic criterion Equation (14) for a porous medium with an axisymmetric loading ($\Sigma_{11}=\Sigma_{22}\neq \Sigma_{33}$; $\Sigma_{12}=\Sigma_{13}\neq \Sigma_{23}=0$) using the friction parameters according to Table 6 are represented in Figure 6.

Yield strain $\varepsilon_{y}$ is the point at which a solid begins to deform permanently under stress, measured as a percentage of the elongation, which in our case coincides with the maximum stress. As can be deduced from the experimental data in Table 4, the samples show a lineal–elastic behavior, which can be describe analytically by the following relationship:

(43) $$\varepsilon_{y}=\frac{\Sigma_{y}}{E}$$

This is typical of a stiff and brittle PLA and could be attributed to its amorphous structure and rigid molecular backbone in its glassy state (below the glass transition temperature, $T_{g}$), which restrict chain mobility and limit plastic deformation. Additionally, high crystallinity, while enhancing rigidity, reduces the polymer’s ability to absorb energy through deformation, leading to brittle failure. The allowable strain for unweathered biocomposites is reduced compared to bulk material due to the existence of voids that act as stress concentrators and reduce the effective cross-sectional area that resists deformation under tensile loading. On the other hand, weathered samples suffer from a significant decrease in the matrix caused by chain scission and embrittlement [70], as well as surface damage or microcracks [108].

### 3.3. Color Change

Raw PLA, initially semitransparent due to its amorphous structure (a very fast cooling rate of injection molding does not allow PLA chains to have conformational capacity for crystallization [109]), exhibits a significant loss of transparency under weathering. This phenomenon is associated with chemo-crystallization driven by chain scission, which enhances chain mobility in amorphous regions and allows partial reorganization into more ordered domains over prolonged xenon arc weathering exposure [82]. However, not all degraded chains undergo crystallization; very short fragments may only form dense amorphous or pseudo-crystalline regions, leading to refractive index variations. These microstructural heterogeneities increase light scattering across the sample thickness, rendering the material more opaque and less glossy, even when the overall increase in crystallinity is moderate. All this results in a darker and less bright surface, which reduces the total reflection and potentially decreases the lightness $L^{*}$ ($\Delta L^{*}<0$) according to Table 7. On the other hand, the color coordinates for PLA_s_ tend towards green ($\Delta a^{*}<0$) and blue ($\Delta b^{*}<0$) with exposure, which is in line with the formation of chromophoric groups and changes in crystallinity.

**Figure 7:**
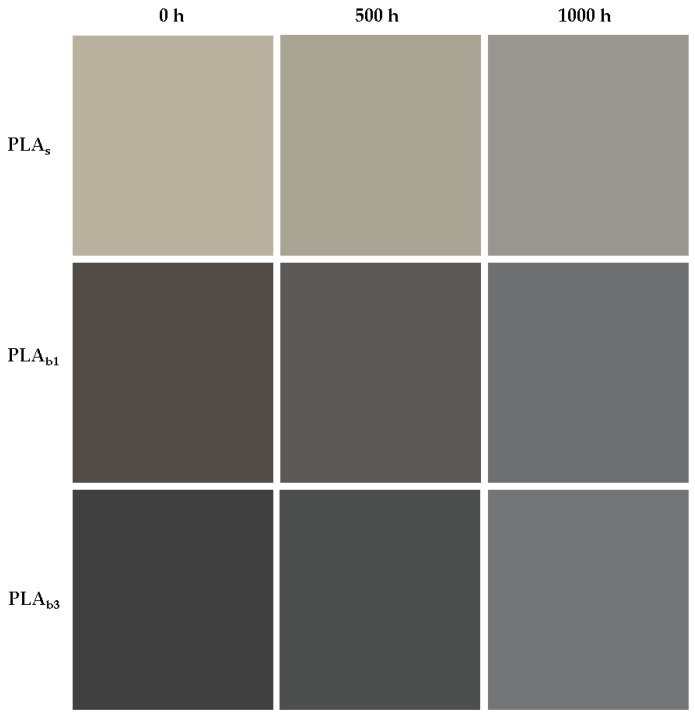
Color according to D65/10° as a function of exposure time for PLA and its biocomposites.

The addition of 1 wt% OBA to the PLA matrix caused a loss of transparency, as well as a darker tone (reduction in $L^{*}$), due to the contribution of ash as a pigment, as can be seen in Figure 7. Regarding the color evaluation of PLA_b1_ during weathering, photo-oxidative reactions result in the formation of chromophores such as carbonyl, hydroxyl, and carboxyl groups, as well as conjugated double bonds [83], causing discoloration; on the other hand, the increase in crystallinity, especially in the amorphous regions, leads to a bleaching effect. This translates quantitatively (Table 7) into a color parameter $L^{*}$ that increases or whitens ($\Delta L^{*}>0$) with weathering time and colorimetric values $a^{*}$ and $b^{*}$ that tend towards green ($\Delta a^{*}<0$) and blue ($\Delta b^{*}<0$), respectively.

When 3 wt% was added into the PLA matrix, the semitransparency feature was completely lost, and the sample appeared totally opaque and darker gray in color in accordance with a simple visual inspection through Figure 7. As with previous biocomposites, for PLA_b3_, the CIELab color space parameters tend towards white ($\Delta L^{*}>0$), green ($\Delta a^{*}<0$), and blue ($\Delta b^{*}<0$).

The quantitative total color parameters $\Delta E^{*}$ in Table 7 reveal that, in general, the most significant change occurred in the values of the $L^{*}$ parameter, which represents lightness. Furthermore, the color change was more substantial in the composites than in the raw polymer. Since the filler/matrix interface facilitates the penetration of UV light, the hydrophilic fillers create pathways for moisture ingress, and the heterogeneous medium increases the fatigue stress due to thermo-mechanical cycling.

### 3.4. Gloss Change

Gloss loss is closely related to surface roughness [21]. UV exposure is a primary driver of gloss loss, as it can break polymer chains and cause surface erosion and microcracks, leading to matting. In addition, moisture absorption promotes hydrolysis (generally more significant in amorphous regions), causing pitting/cracking on the surface and leading to an increase in diffuse reflection and a consequent reduction in gloss. On the other hand, temperature fluctuations and the quality of interfacial adhesion can induce physical cracking and warping.

The addition of ash particles initially reduces the glossiness of the biocomposites compared to neat PLA, as illustrated in Table 8, mainly due to the increased surface roughness introduced by the filler particles. However, during weathering exposure, the biocomposites retained their gloss better than raw PLA, despite the initial differences in lightness between the materials. According to the test results, gloss loss correlates more strongly with the development of surface roughness, microcracks, and filler-induced topography than with color or $L^{*}$ alone, which is consistent with the established understanding that gloss is governed primarily by surface morphology rather than pigment concentration or lightness. In other words, although the lighter color of raw PLA might suggest lower radiation absorption, gloss degradation is more directly determined by surface erosion and topographical evolution than by thermal absorption alone.

### 3.5. FTIR

Polylactic acid is classified as a thermoplastic polyester, which is characterized by repeating monomer units derived from lactic acid [110]. The backbone formula of PLA can be represented as (C_3_H_4_O_2_)_n_ or [–C(CH_3_)HC(=O)O–]_n_, highlighting the arrangement of carbon, hydrogen, and oxygen atoms within the polymer chain [110]. The fundamental building block of PLA is the lactic acid monomer, chemically known as 2-hydroxypropanoic acid (CH_3_CH(OH)COOH), which possesses both a hydroxyl (–OH) and a carboxyl (–COOH) functional group [111]. A key structural feature of PLA is the presence of ester linkages (–COO–) within its polymer chain. The cleavage of these specific bonds during degradation will result in the generation of lactic acid, shorter chain oligomers, and—under complete degradation—carbon dioxide and water [70].

**Figure 8:**
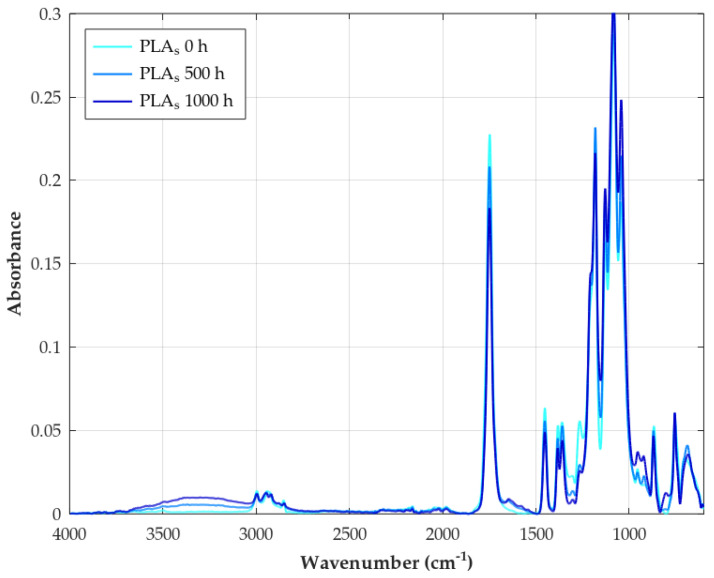
ATR-FTIR spectra of raw PLA specimens before and after weathering.

Unweathered raw PLA exhibits a consistent set of characteristic absorption bands in its FTIR spectrum, which serve as a baseline for identifying the material and detecting any subsequent changes due to degradation. These bands correspond to the vibrational modes of the specific chemical bonds present in the PLA molecule. As can be seen in Figure 8, a prominent band with a peak at $1748\;{\mathrm{cm}}^{-1}$ is observed, which is attributed to the stretching vibration of the carbonyl group (C=O) in the ester linkage [82]. Bands corresponding to the methyl group (CH_3_) are also present, with the asymmetric stretching vibration appearing at $2997\;{\mathrm{cm}}^{-1}$ and the symmetric stretching vibration at $2946\;{\mathrm{cm}}^{-1}\;\;$ [112]. The carbon–oxygen (C–O) stretching vibration of the ester group gives rise to multiple bands in the range of 1040–$1270\;{\mathrm{cm}}^{-1}\;,$ with specific peaks often reported at approximately 1043, 1083, 1128, 1182, 1207, and $1266\;{\mathrm{cm}}^{-1}$ [14]. The bending vibrations of the methyl group are also characteristic, with the asymmetric bending mode at $1452\;{\mathrm{cm}}^{-1}$ and the symmetric bending mode at $1383\;{\mathrm{cm}}^{-1}$ [113]. Additionally, a band around $868\;{\mathrm{cm}}^{-1}$ is often associated with the carbon–carbon (C–C) stretching vibration in the PLA backbone [82]. These characteristic bands collectively form the unique spectral fingerprint of raw PLA, allowing for its identification and providing a benchmark for detecting weathering-induced changes.

During weathering, the chemical structure of PLA changes due to bond cleavage (e.g., ester hydrolysis, photolysis) and the formation of new functional groups (e.g., hydroxyls, carboxylic acids, vinyl groups, hydroperoxides) [114]. These molecular-level alterations manifest directly in the FTIR spectrum (see Table 9) as follows:

- *Decreased ester carbonyl (C=O) stretching:* An absorption peak observed at $1748\;{\mathrm{cm}}^{-1}$ corresponds to the stretching vibration of the ester carbonyl group (C=O) in the polymer backbone [82]. A significant reduction in the intensity of this band is a primary and universal indicator of PLA degradation during weathering, reflecting the cleavage of ester bonds via hydrolysis and photolytic chain scission [115].
- *Appearance of hydroxyl (O–H) stretching:* Weathering leads to the formation or accumulation of hydroxyl groups, resulting in the appearance of broad absorption bands in the high-frequency region, typically ∼3100–$3700\;{\mathrm{cm}}^{-1}$ [59]. These bands are assigned to the O–H stretching vibrations of carboxylic acid end groups (–COOH) and alcohol end groups (–OH) formed by hydrolysis, as well as potentially to hydroperoxides (–OOH) formed during photo-oxidation processes [116].
- *Appearance of carbon–carbon double bonds (C=C):* A key indicator of the Norrish Type II photolysis mechanism is the appearance of new absorption peaks in the region 1645–$1650\;{\mathrm{cm}}^{-1}\;\;$ These bands are assigned to the stretching vibration of C=C double bonds, specifically the vinyl end groups formed as a direct product of this photochemical chain scission pathway [80].
- *Changes in C–O stretching region:* The spectral region between approximately 1000 cm^−1^ and $1300\;{\mathrm{cm}}^{-1}$ is complex, containing multiple overlapping bands associated with various C–O stretching vibrations within the PLA structure (e.g., asymmetric and symmetric C–O–C stretches) as well as C–C stretches. Characteristic peaks for PLA are identified at 1266, 1208, 1182, 1129, 1083, and $1043\;{\mathrm{cm}}^{-1}\;\;$ Degradation causes noticeable changes in this region, for example, peaks at $1182\;{\mathrm{cm}}^{-1}$ have been linked specifically to ester bond cleavage during hydrolysis/oxidation, lowering absorbance [117]. Decreases in intensity at $1266\;{\mathrm{cm}}^{-1}$ and changes around $1129\;{\mathrm{cm}}^{-1}$ have also been observed during thermal/hydrolytic aging. Conversely, increases at $1043\;{\mathrm{cm}}^{-1}$ and $1208\;{\mathrm{cm}}^{-1}$ (attributed to new C=O formation) were noted during thermo-oxidative degradation [118].
- *Changes in C–H region:* Bands related to C–H vibrations include asymmetric or symmetric stretching (∼2800–$3000\;{\mathrm{cm}}^{-1}$) and bending or deformation modes (e.g., methyl group bends at $1452\;{\mathrm{cm}}^{-1}$ and $1359\;{\mathrm{cm}}^{-1}\;,$ C–H deformations at $1383\;{\mathrm{cm}}^{-1}$) [119]. The band around $1455\;{\mathrm{cm}}^{-1}$ (CH deformation or bending) is often considered relatively stable and is sometimes used as an internal reference peak for calculating degradation indices [59]. Reduced intensity in the $1359\;{\mathrm{cm}}^{-1}$ region has also been linked to thermo-oxidative degradation [118].
- *Changes in crystallinity-related bands:* Specific bands in the fingerprint region can be sensitive to the polymer’s morphology (crystalline vs. amorphous phases). A peak around $921\;{\mathrm{cm}}^{-1}$ is often associated with ordered structures or α-crystals, while a peak near $955\;{\mathrm{cm}}^{-1}$ is attributed to the amorphous phase [118]. Changes in the relative intensities of these bands during weathering can indicate an increase in crystallinity (chemo-crystallization).

**Figure 9:**
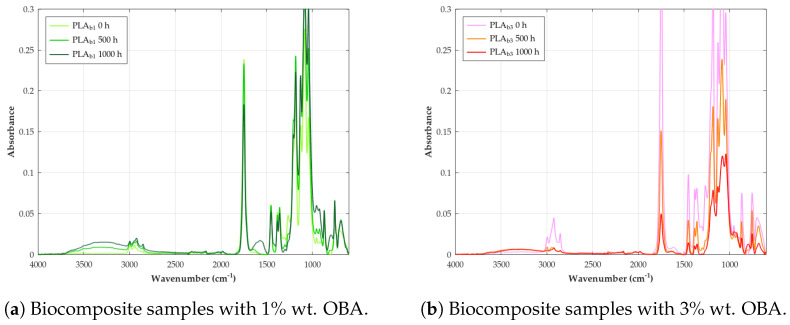
ATR-FTIR spectra before and after weathering.

As far as biocomposites are concerned, we can deduce from Figure 9 that filler incorporation does not introduce new chemical bonds (no significant new peaks), suggesting that physical rather than chemical interactions dominate. In order to quantify the change in intensity bands between the FTIR spectra of raw PLA and its biocomposites, we have made use of two metrics widely known in this context, which are CI and HI [58,59,80]. Indexes are calculated as the specified area under band (SAUB), resulting in the values from Table 10. From this, we can observe an unclear trend for the CI, while the HI always decreases regardless of the material grade.

The complex changes taking place in the carbonyl groups can be explained by the fact that the C=O stretching band is already present in the matrix before the weathering [120], the low formation of new carbonyls due to oxidation [115], and the slow hydrolysis of the ester bonds [16]. On the other hand, there is the HI increase with the exposure time due to the formation of –OH end groups from hydrolysis and potentially hydroperoxides or alcohol groups from photo-oxidation [116]. This effect is even more pronounced the higher the OBA content because of the hydrophilic character of the filler, which can absorb and retain moisture, accelerating hydrolytic degradation of the PLA matrix [121].

Understanding the degradation process is not only vital for predicting the longevity of PLA and its biocomposites in various applications but also for evaluating its true environmental impact [122]. FTIR spectroscopy, with its ability to probe the molecular vibrations of chemical bonds, offers a unique window into these structural changes, allowing for the identification of specific degradation products and the monitoring of the degradation process over time.

### 3.6. SEM

**Figure 10:**
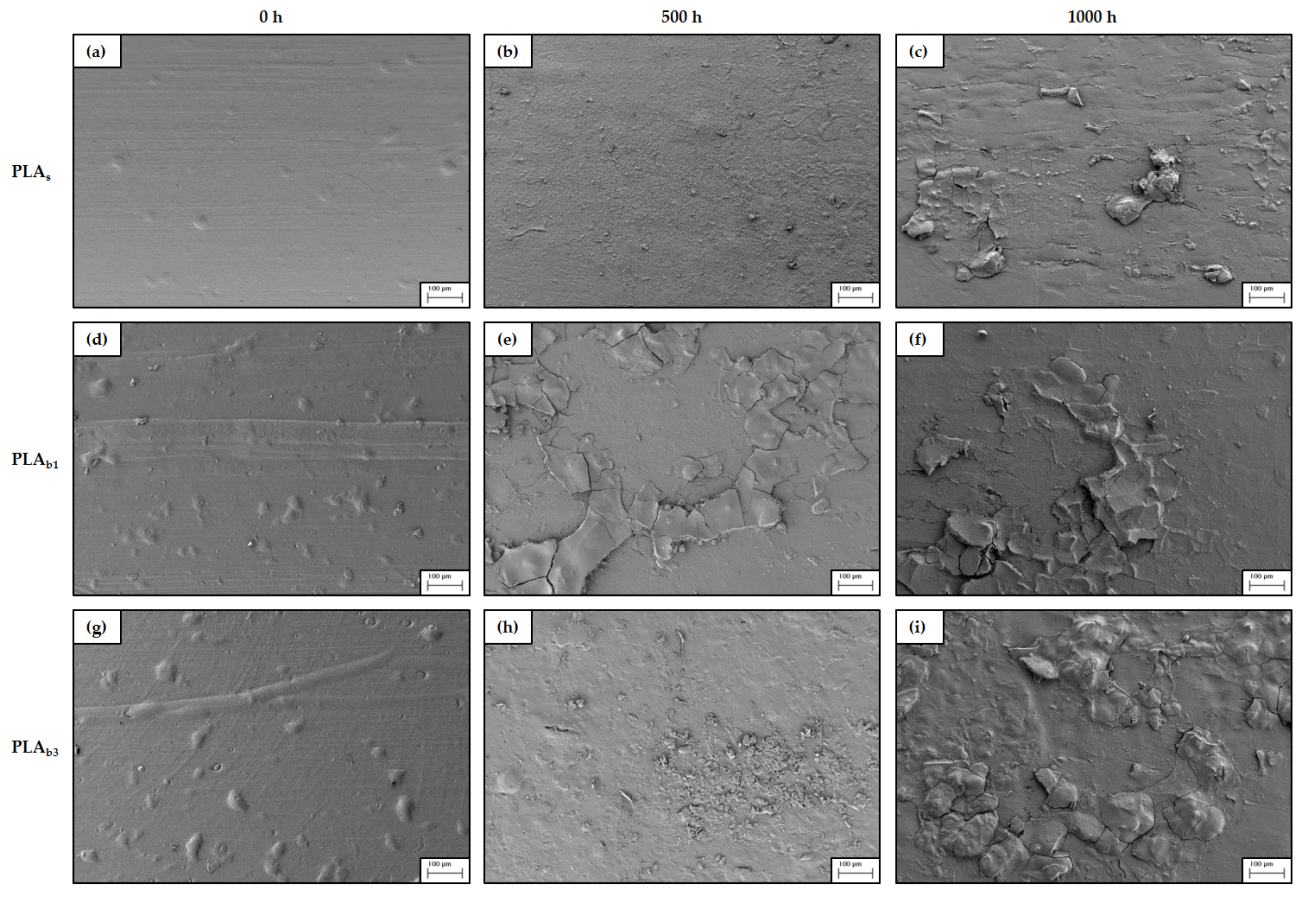
SEM images of the surface before and after weathering (Mag. 100×): (**a**) PLA_s_ 0 h; (**b**) PLA_s_ 500 h; (**c**) PLA_s_ 1000 h; (**d**) PLA_b1_ 0 h; (**e**) PLA_b1_ 500 h; (**f**) PLA_b1_ 1000 h; (**g**) PLA_b3_ 0 h; (**h**) PLA_b3_ 500 h; (**i**) PLA_b3_ 1000 h.

**Figure 11:**
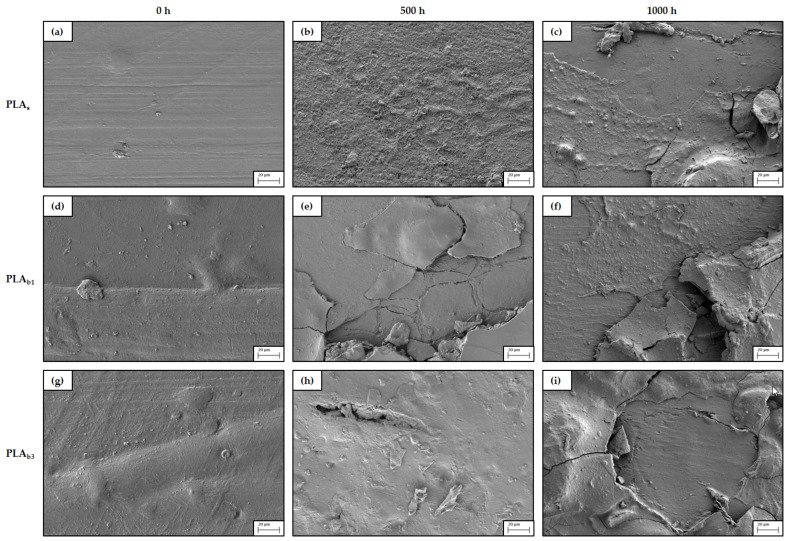
SEM images of the surface before and after weathering (Mag. 500×): (**a**) PLA_s_ 0 h; (**b**) PLA_s_ 500 h; (**c**) PLA_s_ 1000 h; (**d**) PLA_b1_ 0 h; (**e**) PLA_b1_ 500 h; (**f**) PLA_b1_ 1000 h; (**g**) PLA_b3_ 0 h; (**h**) PLA_b3_ 500 h; (**i**) PLA_b3_ 1000 h.

Weathering can significantly alter the surface morphology and physical integrity of raw PLA and its biocomposites. Scanning Electron Microscopy (SEM) is an indispensable tool for visualizing these changes at the microscale that take place on the surface exposed during weathering. Initially (Figure 10a and Figure 11a), the raw PLA has a uniform and smooth surface that is relatively featureless, with the exception of a few defects, such as (a) grooves left in the mold by the milling cutter during manufacture and (b) small irregularities due to wear caused by the repeatability of the injection cycle. On the other hand, the protrusions grow in number and size in Figure 10d,g and Figure 11d,g, which display the typical features of composite systems, where one can easily recognize a dispersed phase wrapped in a polymer, resulting in a slightly rougher surface than the PLA matrix alone.

After 500 h, the raw PLA (Figure 10b) presents a rather rough texture and starts to show signs of surface erosion consistent with the expected chemical degradation (photolysis/hydrolysis). Higher magnification (Figure 11b) reveals the existence of uneven porosity with microcavities or pitting, as well as microcracks of fissures. On the other hand, for the biocomposites (Figure 10e,h), there is already flaking at this time point, suggesting that the presence of fillers causes stress concentration that facilitates crack initiation and propagation. This effect is even more relevant for the PLA with a higher ash content, since while in Figure 11e, some cracks and partial peeling can be seen, in Figure 11h, a practically complete delamination of the topmost layer can be noticed, as well as deeper fractures.

For longer exposure, 1000 h, the topography of the PLA_s_ samples (Figure 10c and Figure 11c) show layer fragmentation and extensive cracking; these features are consistent with embrittlement due to chain scission and with thermo-mechanical stresses associated with cyclic temperature and humidity [123]. In reference to the biocomposites, the high-resolution images in Figure 10f,i and Figure 11f,i illustrate cumulative damage and more severe degradation, pointing to further loss of material. Advanced degradation with pronounced cracks, delamination, significant erosion, and porosity indicates progressive damage from prolonged exposure [124].

### 3.7. DSC

Given that weathering processes induce chemical and physical changes in polymers, such as chain scission, crosslinking, and changes in morphology (crystallinity), DSC is an essential tool to monitor phase transitions and thermal reactions, alongside enthalpic changes [125]. Changes in glass transition ($T_{g}$), cold crystallization ($T_{cc}$), and melting ($T_{m}$), as well as the degree of crystallinity ($\chi_{c}$) measured by this type of analysis, can provide valuable insights into the extent and nature of the degradation occurring in PLA and its biocomposites upon exposure to weathering conditions.

The first transformation phenomenon in the DSC thermogram is the $T_{g}$, where the material transitions from a glassy to a rubbery state. At constant pressure, this can essentially be translated into an inflection point for the heat capacity ($C_{p}$) curve—calculated with the first derivate of the enthalpy with respect to temperature (dH/dT)—roughly in the range estimated by the material supplier (55–60 °C). For unfilled PLA, as can be seen in Table 11, a slight increase in $T_{g}$ is observed after weathering. This change is consistent with chemo-crystallization and physical aging in the amorphous phase. Chain scission in amorphous regions generates shorter segments that can reorganize and increase crystallinity, restricting segmental mobility and thus requiring more thermal energy to initiate the glass transition or a modest $T_{g}$ rise [126]. Therefore, the slight net increase in $T_{g}$ for raw PLA indicates that, under the present exposure conditions, crystallinity build-up and physical aging dominate over the $T_{g}$-lowering effect of chain scission, which is consistent with the concurrent increase in $\chi_{c}$ obtained from DSC [127]. Conversely, the incorporation of OBA exhibiting a downward trend of $T_{g}$, which might be motivated by (a) promotion of moisture absorption due to its hydrophilic feature, resulting in an acceleration of hydrolytic degradation [128]; (b) insufficient interfacial adhesion between the hydrophobic matrix and the hydrophilic fillers [129]; and (c) increased photodegradation caused by a higher radiation absorption of the biocomposite surface due to its darker color.

**Figure 12:**
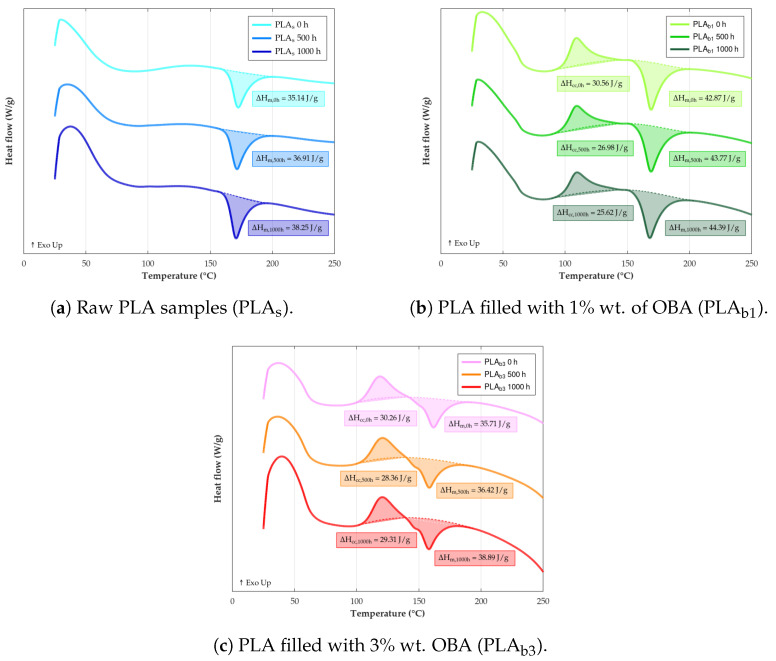
DSC heat flow curves (2nd run, 10 °C/min) of PLA and its biocomposites at different time points.

After this, if a semicrystalline polymer is heated above its $T_{g}$, the chains may gain sufficient mobility to crystallize before melting; this is called “cold crystallization” and appears as an exothermic peak during the heating scan. The crystallization pattern and consequently its thermal properties could be modified by the incorporation of filler into the PLA matrix, as can be deduced form the DSC graphs shown in Figure 12, where it is observed that while the composites undergo the crystallization process, neat PLA shows no distinct $T_{cc}$ peak under the applied heating protocol, despite a measurable increase in $\chi_{c}$. The difference in behavior between both material variants for a temperature below the melting temperature may be linked to two main factors: (a) relatively high initial crystallinity and additional crystallization during weathering, mainly via slow chemo-crystallization at service temperature and during the first heating ramp, yielding a small enthalpy that is distributed over a broad temperature range and not resolved as a sharp $T_{cc}$ peak for PLA_s_ [130], and (b) the nucleation effect of fillers in the PLA_b_, which could provide surfaces or sites that promote the formation of crystals [131,132]. Regarding the evolution of the $T_{cc}$ temperature of the composites as the exposure time elapses, it is possible to notice a slight enhance linked to the fact that the volume of the amorphous phase available to undergo cold crystallization is already significantly reduced while degradation occurs [133].

The slightly higher $T_{g}$ observed for PLA_b1_ (63.2 °C) compared to typical PLA values (55–60 °C) can be attributed to a combination of factors. First, PLA is highly sensitive to physical aging, and chain densification during storage or processing increases the mobility restriction in the amorphous phase, shifting $T_{g}$ upward. Second, the nucleating action of OBA may induce a subtle increase in initial crystallinity, which reduces amorphous fraction mobility. Finally, DSC first-heating scans reflect the material’s thermal history, amplifying these effects. Therefore, the elevated $T_{g}$ does not indicate anomalous behavior but is fully consistent with semicrystalline PLA systems containing nucleating fillers [134].

A second endothermic transition appears in the DSC thermogram if heating goes on, which is associated with the intermolecular forces that hold the crystal lattice together, resulting in a breakdown of the ordered crystalline structure, whereby the matrix is transformed into a disordered, viscous (molten) liquid. The observed decrease in melting temperature ($T_{m}$) is likely caused by the destruction of crystalline perfection. Photodegradation interrupts the regular lamellar structure and induces defects within the crystal lattice. While the reduction in average molecular weight contributes to this effect, the dominant factor is the formation of smaller, less perfect crystallites which melt at lower temperatures [135]. On the other hand, the addition of OBA introduces interfacial regions and geometric constraints that can locally hinder lamellar thickening and perfection, contributing to a reduction in $T_{m}$. At the same time, their surfaces act as heterogeneous nucleation sites, as reflected by the appearance/intensification of cold crystallization peaks and the progressive increase in $\chi_{c}$ with weathering time. Thus, OBA behaves as a nucleating filler that increases the number of crystals but may limit crystal perfection and lamellar growth [136,137].

The decrease in melting temperature observed for PLAb3 (158–161 °C) is explained by the formation of imperfect and thinner lamellae. The higher OBA content promotes heterogeneous nucleation at particle agglomerates, generating a larger number of small crystalline domains with reduced lamellar thickness, which melt at lower temperatures [138]. Additionally, the photolytic and hydrolytic degradation processes documented through CI, HI, and crack-density measurements contribute to reduced molecular weight, further limiting crystal perfection. As a result, PLA_b3_ develops less stable crystalline structures, which is consistent with its lower $T_{m}$ and greater overall degradation.

Beyond identifying transition temperatures, DSC provides quantitative information through the measurement of enthalpy changes associated with these transitions (see Figure 12). The area under the melting peak in a heating scan corresponds to the enthalpy of fusion or melting ($\Delta H_{m}$), representing the amount of heat absorbed per unit mass of sample to melt the crystalline portion. Similarly, the area under a crystallization peak corresponds to the enthalpy of crystallization ($\Delta H_{cc}$), representing the heat released during crystal formation [125]. To calculate the degree of crystallinity ($\chi_{c}$) of the sample, the enthalpy of cold crystallization must be subtracted from the melting enthalpy and relativized to 100% crystalline PLA and, in the case the compounds, adjusted to take into account the weight fraction of ash, resulting in the values given in Table 11. From these numbers, we can conclude what we anticipated previously through the transition points of the diagram as follows: (a) in the case of unweathered samples, the incorporation of OBA slightly reduces the overall crystallinity compared with neat PLA, which can be attributed to restricted chain mobility and the presence of rigid inclusions that interfere with lamellar growth during fast injection mold cooling; (b) during weathering, OBA acts predominantly as a heterogeneous nucleating agent, since, although agglomerated particles may locally constrain lamellar growth, the dominant effect in our system is nucleation, which increases the number of crystallization sites and promotes the formation of smaller, more numerous crystalline domains.

### 3.8. Kinetics

Thermogravimetric analysis (TGA) is a fundamental analytical technique to assess the thermal stability and decomposition behavior of PLA and its biocomposites. Alterations in TGA profiles, such as changes in degradation onset temperature or changes in mass loss stages, indicate variations in thermal stability and degradation pathways [139]. As a complement to TGA, the study of reaction kinetics provides quantitative information on energy barriers (e.g., activation energy) and mechanisms governing thermal decomposition [65].

**Figure 13:**
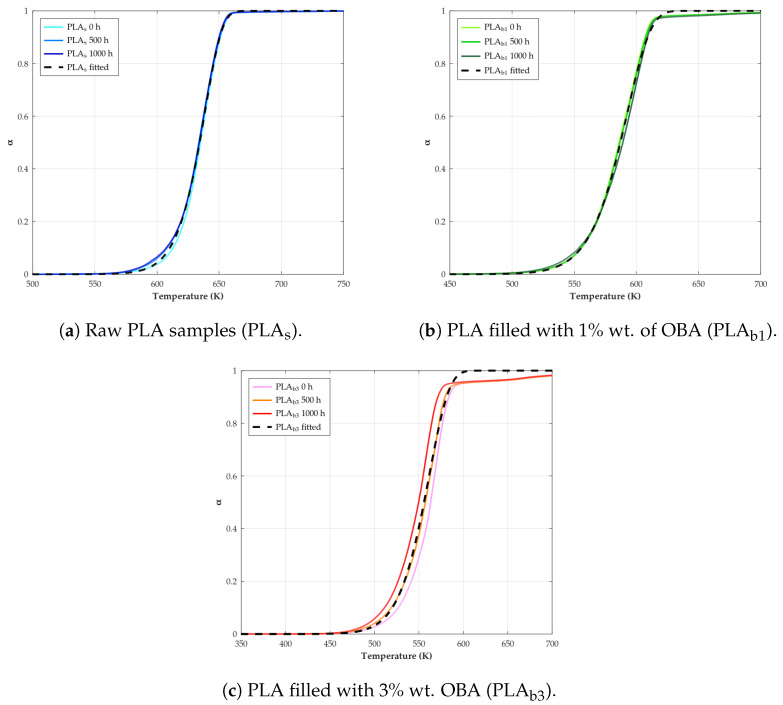
Degree of conversion curves at different time points and fitting models of PLA and its biocomposites.

Thermal degradation of solid-state polymers is a complex process involving a variety of chemical reactions, such as chain scission, depolymerisation, and side group removal. However, parameters such as the kinetic triplet ($E_{a}$, *A*, and the form of f[α]) can provide a mathematical framework to describe the rate of these processes and their temperature dependence [140,141]. These key parameters are determined by model fitting analyzing the TGA data, which involves assuming a particular reaction model f[α] and then fitting the experimental TGA data to the equation of the chosen model to determine the activation energy ($E_{a}$) and the pre-exponential factor (*A*). Of all the reaction models tried for the fit—Power law ($P_{rm}$), Diffusion ($D_{rm}$), Mampel ($F_{rm}$), Contracting ($R_{rm}$), and Avrami–Erofeev ($A_{rm}$) [142]—it is the latter that has shown the best goodness of fit to the experimental data (see Figure 13).

Although the Avrami–Erofeev model was originally developed for solid-state crystallization (KJMA framework), its sigmoidal form has been widely applied as a phenomenological description of autocatalytic decomposition processes in polymers [143]. In this work, the model is used empirically to reproduce the shape of the conversion curves and to extract an “apparent” kinetic triplet rather than to claim a specific microscopic nucleation–growth mechanism for PLA degradation. The equation defining this mechanism and its integral form can be expressed generically as follows:

(44) $$f\left[\alpha \right]=n\left(1-\alpha \right){\left(-\ln\left(1-\alpha \right)\right)}^{\frac{n-1}{n}}$$

(45) $$g\left[\alpha \right]={\left(-\ln\left(1-\alpha \right)\right)}^{\frac{1}{n}}$$

where *n* is the Avrami exponent, which is a dimensionless parameter that provides insights into the nucleation mechanism and the dimensionality of growth. Equation (45), in combination with Equation (24), allows us to represent the extent of conversion explicitly as a function of temperature:

(46) $$\alpha \left[T\right]=1-\exp\left(-\frac{A}{\beta }{\left(Te^{\frac{-E_{a}}{RT}}-T_{0}e^{\frac{-E_{a}}{RT_{0}}}-\frac{E_{a}}{R}\left(E_{i}\left[\frac{E_{a}}{RT}\right]-E_{i}\left[\frac{E_{a}}{RT_{0}}\right]\right)\right)}^{n}\right)$$

The kinetic triplet as an average of the three time points is given in Table 12 for each material. The apparent activation energy obtained from global fitting decreased with increasing OBA content, while the pre-exponential factor also showed a downward trend. These changes indicate that ash addition modifies the effective energy barrier and frequency of thermally activated events during decomposition; however, distinguishing between contributions from catalytic effects, plasticization, structural defects, or altered chain mobility is not possible from TGA alone [144,145]. On the other hand, with respect to the Avrami exponent, n≈1.5 for raw PLA suggests instantaneous nucleation with diffusion-controlled 3D growth or sporadic nucleation with diffusion-controlled 1D growth, while n≈1 for biocomposites suggests instantaneous nucleation with diffusion-controlled 2D growth or interphase-controlled 1D growth [146].

**Figure 14:**
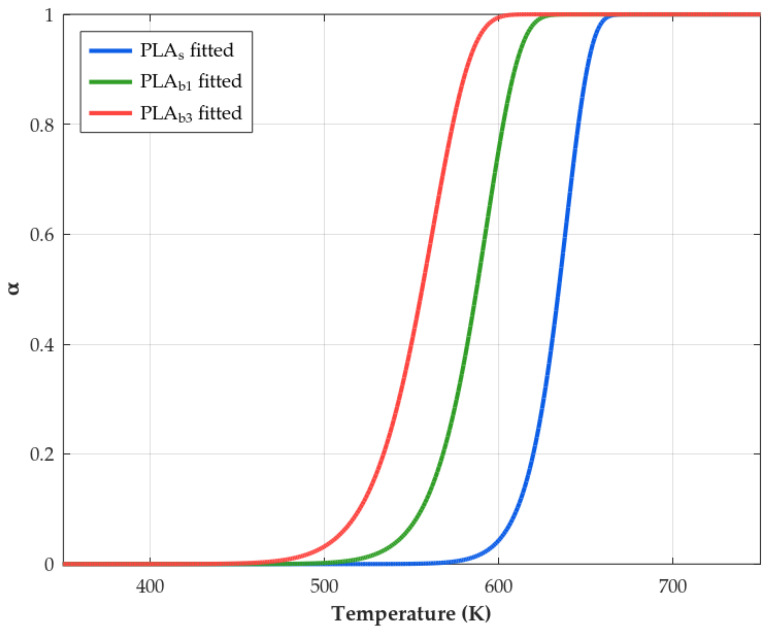
Thermal decomposition of raw PLA and its biocomposites evaluated by TGA at a heating rate of 10 °C/min under nitrogen atmosphere.

Hydrophilic components like natural fillers often accelerate hydrolytic degradation by increasing moisture uptake, leading to more pronounced reductions in thermal stability, as can be seen in Figure 14, where the decomposition temperatures of the biocomposite gradually decrease with increasing ash content. This reduction in thermal stability might be attributed to a catalytic effect of OBA, particularly due to its potassium content, which accelerates PLA matrix degradation. Catalytic action may involve a hydrolytic reaction mechanism facilitated by residual hydroxyl groups on the filler surface [145]. Furthermore, rather than attributing the behavior of the biocomposites to a generic reduction in cohesion energy, the results indicate that the incorporation of OBA leads to a heterogeneous morphology with weak interfacial adhesion. Such interfacial discontinuities act as preferential pathways for moisture and heat transport, which increase, as revealed by SEM images, the formation of surface defects and microvoids [147]. This enhanced permeability accelerates hydrolytic and photochemical chain scission, which is consistent with the reduced thermal stability observed in TGA and with the progressive decay of tensile modulus and yield strength.

**Figure 15:**
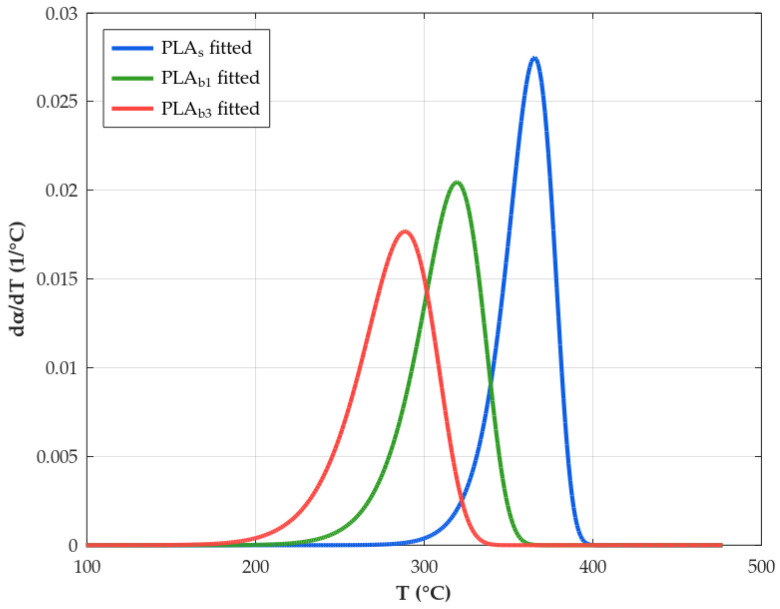
Thermal decomposition of raw PLA and its biocomposites evaluated by DTGA at a heating rate of 10 °C/min under nitrogen atmosphere.

The thermal stability of the unweathered composites was evaluated to assess the influence of the biomass ash on the PLA matrix. As detailed in Table 13 and Figure 15, the addition of OBA resulted in a noticeable reduction in thermal stability. The onset degradation temperature ($T_{onset}$) decreased from 329 °C for raw PLA to 271 °C and 234 °C for the 1 wt% and 3 wt% composites, respectively.

This behavior aligns with recent findings [148], which demonstrated that certain inorganic fillers can catalyze the thermal degradation of polylactide. In the case of OBA, this catalytic effect is likely attributed to the presence of alkaline earth metal oxides (such as CaO and K_2_O) inherent in olive stone biomass ash, which can promote random chain scission and hydrolysis mechanisms at elevated temperatures.

Despite this reduction in thermal stability, it is important to note that the $T_{onset}$ of the composite with the highest loading (PLA_b3_, 234 °C) remained significantly higher than the processing temperature used for these biocomposites (190 °C). This confirms that while the processing window is narrower compared to neat PLA, the material retains sufficient thermal stability to be manufactured without undergoing degradation during the extrusion and injection molding cycles.

### 3.9. Acceleration Factor

The acceleration factor (AF) is theoretically defined as the ratio of the time required to reach a certain degradation level under natural conditions to the time required under accelerated conditions [149]. Ideally, this definition assumes that the degradation mechanisms remain identical between both environments. However, it must be noted that accelerated weathering, with its higher intensity and controlled cycles, can potentially introduce specific degradation pathways (e.g., radical formation rates or thermal effects) that may differ from real service conditions, particularly for complex biopolymers sensitive to UV and moisture.

The use of an optically filtered xenon arc lamp provides a spectral distribution that closely simulates natural sunlight [66]. Based on this approximation, an energy-based calculation for the partial acceleration factor due to light ($AF_{light}$) can be estimated. While this approach simplifies the calculation by assuming wavelength independence, it is important to recognize that the specific spectral sensitivity of the composite-influenced by the polylactic acid matrix and the OBA fillers may lead to selective absorption that a purely energy-based model does not fully capture. Regarding the photochemical kinetics, the degradation process is often modeled using the classical reciprocity law (Bunsen–Roscoe law), which implies that the degree of degradation depends solely on the total absorbed energy (p=1) [150]. Although this study adopts p=1 for the estimation of AF (Equation (27)), deviations from this law are frequently observed in biodegradable polymers, where limited oxygen diffusion or complex oxidation mechanisms may alter the effective reaction order.

Furthermore, PLA is a condensation polymer containing ester linkages susceptible to hydrolysis. To account for the contribution of moisture, a kinetic model incorporating relative humidity (RH) is used. A second-order dependence on humidity (n=2) is often proposed for hydrolysis in polyesters [151] and is applied here as a standard kinetic approximation (Equation (31)). It is acknowledged, however, that the exact value of the exponent *n* varies depending on the specific material composition and filler interactions, and determining the precise value for these OBA-reinforced biocomposites would require extensive experimental validation.

(47) $$AF_{total}=AF_{light}\times AF_{temp}\times AF_{hum}=\frac{H_{acc}}{H_{out}}e^{\frac{E_{a}}{R}\left(\frac{1}{T_{out}}-\frac{1}{T_{acc}}\right)}{\left(\frac{RH_{acc}}{RH_{out}}\right)}^{2}$$

According to ISO 877, two benchmark regions used for exposure tests on plastics are South Florida (Miami) and the mid-Arizona desert (Phoenix), which correspond to tropical savannah (Aw) and subtropical desert (BWh) climates, respectively [152]. The annual TUV accumulation solar radiation in these areas is given by Table 2 for 5 and 45 tilt angles. Based on the parameters set in the weathering chamber, the radiation emitted by the xenon lamp is $0.51\;W/(m^{2}\;\cdot \;\mathrm{nm})$ at 340 nm, which is equivalent to an averaged irradiance of 60 W/m^2^ following the cycle 1 from the ISO 4892-2. Regarding the effective temperature, it will be depend on the sample color [21], being for clear specimens similar close to air temperature while for black being higher due to greater energy absorption.

As can be deduced from Table 14, while the average annual radiation level and temperature are quite close in both outdoor locations, there is a significant difference in the humidity load in the air, which makes the Miami climate a much harsher environment for our material due to the hydrolysis process. Combining the thermal, moisture, and irradiance contributions, the laboratory test conditions are theoretically estimated to yield an acceleration factor of at least 15 compared to the field test conditions (based on solar energy and average RH). This value serves as an initial approximation. A rigorous determination of the AF would require long-term field data to validate the extent to which the synergistic effects of photo-, hydro-, and thermodegradation in the laboratory correlate with natural weathering for this specific biocomposite system.

## 4. Conclusions

This study investigated the effects of incorporating OBA as a reinforcement filler in PLA biocomposites, focusing on their performance under accelerated weathering conditions. PLA biocomposites with varying OBA contents (0%, 1%, and 3% wt.) were subjected to comprehensive characterization, including mechanical performance, thermal behavior, structural changes, morphology, and surface appearance, both immediately after manufacture (0 h) and after accelerated weathering (500 h and 1000 h). The key findings are summarized as follows:

- *Weathering resistance:* OBA reinforcement accelerated the degradation of PLA biocomposites under UV, heat, and humidity cycles. Higher OBA content led to more severe surface erosion, microcracking, and delamination due to poor filler–matrix interfacial adhesion and moisture absorption by hydrophilic OBA.
- *Mechanical properties:* The addition of OBA reduced the stiffness and tensile strength of PLA, with greater reductions at higher filler loadings. This is attributed to OBA agglomerates acting as a soft inclusion (stress concentrator) and weak interfacial bonding, as confirmed by micromechanical modeling and SEM analysis. Weathering further reduces mechanical properties by inducing chain scission and microcracks. Crack density increased with exposure time and was highest in PLA_b3_.
- *Optical and aesthetic properties:* Weathering led to noticeable changes ($\Delta E^{*}$) in color and surface gloss. While raw PLA darkened and lost gloss due to crystallization and surface erosion, biocomposites showed a whitening effect and better gloss retention, albeit starting from a lower initial gloss due to ash content.
- *Chemical degradation:* FTIR analysis revealed that weathering caused ester bond cleavage, the formation of hydroxyl and carbonyl groups, and increased crystallinity. The HI increased significantly with exposure time, especially in ash reinforced samples, indicating enhanced hydrolytic degradation. The CI showed no consistent trend, likely due to competing degradation mechanisms (photolysis vs. ester bond cleavage).
- *Thermal behavior:* OBA acted as a nucleating agent, promoting cold crystallization in PLA and increasing crystallinity ($\chi_{c}$) over weathering time. However, it lowered the melting temperature ($T_{m}$) and glass transition temperature ($T_{g}$), indicating reduced thermal stability. Kinetics analysis revealed lower activation energy ($E_{a}$) and pre-exponential factor (*A*) values for biocomposites, confirming accelerated thermal degradation with OBA addition. This is potentially due to the catalytic effect of OBA’s potassium content and poor filler–matrix cohesion.
- *Morphological changes:* SEM observations indicated that weathering induced surface microcracking and roughness, which were more severe in PLA_b3_, suggesting that higher filler content exacerbates surface degradation. SEM confirmed advanced surface degradation in OBA composites, including pitting, flaking, and delamination after 1000 h of exposure.
- *Acceleration Factor (AF):* The xenon arc accelerated weathering protocol (ISO 4892-2) provided an AF of ≥15× relative to natural weathering in Miami or Phoenix, where humidity (hydrolysis) was a critical degradation driver, especially for biocomposites. However, it is emphasized that this is an estimate and not an absolute value.

In conclusion, while OBA valorizes agricultural waste and supports sustainability goals, its incorporation into PLA biocomposites requires careful optimization for long-term durability under environmental stress. The size of the filler should be reduced to the nanoscale to avoid agglomeration and deterioration of the mechanical properties, and surface treatments (e.g., silanes, polymeric compatibilizers) or barrier elements should be used to improve compatibility with PLA and prevent moisture absorption. These findings contribute to the development of sustainable biocomposites by underscoring the need for tailored material formulations to ensure durability in real world applications.

Based on the analyzed results, although the addition of olive pit ash resulted in a reduction in tensile strength or thermal stability, the material demonstrated acceptable aesthetic and barrier properties. Consequently, these biocomposites are not suitable for high-load structural applications. Instead, they are promising candidates for low-load devices and eco-friendly applications. Potential uses include packaging for agricultural or horticultural products, as well as disposable garden or nursery accessories, where the biodegradability of the matrix is preserved, and the use of ash reduces the overall cost and carbon footprint of the final product.

Future work will involve measuring molecular weight to quantify degradation and damage evolution to define constitutive models of materials. In addition, natural weathering tests will be carried out in order to correlate outdoor and laboratory tests, as well as to achieve a more accurate acceleration factor estimation.

## Figures and Tables

**Table 1:** Data sheet for Piropel Mix Biomasud A1 OP (report from CARTIF Technology Center).

Parameter	Method/Standard	Value	Uncertainty	Units
Total humidity	UNE-EN ISO 18134-1:2023	10.8	±0.3	% m/m ^(1)^
Ashes	ISO 18122:2022	0.6	±0.2	% m/m ^(2)^
Bulk density	UNE-EN ISO 17828:2016	780	±40	kg/m^3 (1)^
Oil and fat content *	Internal procedure based on UNE EN ISO 659	0.31	–	% m/m ^(1)^
Elemental Analysis				
Carbon	UNE-EN ISO 16948:2015 Instrumental method	50.3	±3.5	% m/m ^(2)^
Hydrogen		6.5	±0.9	% m/m ^(2)^
Nitrogen		0.28	±0.04	% m/m ^(2)^
Sulfur	UNE-EN ISO 16994:2017	0.024	±0.008	% m/m ^(2)^
Chlorine	Combustion method and ion chromatography	0.019	±0.007	% m/m ^(2)^
Oxygen *	Calculated	42.4	–	% m/m ^(2)^
Energy Analysis				
Gross Calorific Value corrected to constant volume (GCV_V_)	UNE-EN ISO 18125:2018	20.9	±0.4	MJ/kg ^(2)^
Net Calorific Value to constant pressure (NCV_P_)		19.5	±0.4	MJ/kg ^(2)^
		17.2	±0.9	MJ/kg ^(1)^
		4.8	±0.3	kWh/kg ^(1)^

* The test results and the statement of compliance with the specification in this report refer only to the test specimen. The BIOMASUD classification has been made without taking into account the measurement uncertainty, following indications of the control and certification bodies, and may have a specific risk of false acceptance of up to 50%. ^(1)^ According to sample received. ^(2)^ Dry base.

**Table 2:** Average annual total UV (TUV, 295–385 nm) solar radiation, temperature (*T*), and relative humidity (RH) for south-facing exposures reported at commercial exposure sites (2000–2023).

Exposure Site	Miami		Phoenix	
Tilt angle ^1^	$5^{\circ }$	$45^{\circ }$	$5^{\circ }$	$45^{\circ }$
TUV (MJ/m^2^)	340 ± 16	325 ± 17	376 ± 32	366 ± 30
T (°C)	24.4 ± 1.2		24.3 ± 1.7	
RH (%)	79.5 ± 2.9		33.8 ± 5.3	

^1^ From horizontal. Weather data from Q-Portal Web Tools (https://www.q-portal.net/Applications/QPortal/LoadUserControl.aspx?user_control_name=mwt_weather_data&unique_id=null) (accessed on 16 May 2025) (Q-lab).

**Table 3:** Mass of raw PLA and its biocomposites before (initial) and before (final) weathering.

Material	Exposure Time (h)	Conditioned Mass (g)		Dry Mass ^1^ (g)	
		Initial	Final	Initial	Final
PLA_s_	0	1.2899 ± 0.0030	-	1.2884 ± 0.0030	-
	500	1.2902 ± 0.0021	1.2893 ± 0.0021	1.2886 ± 0.0021	1.2823 ± 0.0021
	1000	1.2897 ± 0.0039	1.2904 ± 0.0037	1.2881 ± 0.0039	1.2821 ± 0.0037
PLA_b1_	0	1.2951 ± 0.0041	-	1.2853 ± 0.0040	-
	500	1.2916 ± 0.0023	1.2930 ± 0.0018	1.2819 ± 0.0023	1.2792 ± 0.0017
	1000	1.2983 ± 0.0041	1.2939 ± 0.0034	1.2885 ± 0.0041	1.2777 ± 0.0034
PLA_b3_	0	1.3175 ± 0.0038	-	1.2565 ± 0.0036	-
	500	1.3173 ± 0.0041	1.3162 ± 0.0036	1.2563 ± 0.0039	1.2257 ± 0.0034
	1000	1.3169 ± 0.0039	1.3078 ± 0.0081	1.2558 ± 0.0038	1.1990 ± 0.0075

^1^ Values obtained considering the weight reduction that takes place during heating up to 200 °C in the 1st TGA phase.

**Table 4:** Tensile results for raw PLA and its biocomposites after accelerated weathering test.

Material	Exposure Time (h)	Bulk Mechanical Properties		
		E (GPa)	$\Sigma_{y}$ (MPa)	$\varepsilon_{y}$ (%)
PLA_s_	0	3.48 ± 0.11	62.4 ± 0.6	1.79 ± 0.05
	500	3.41 ± 0.08	51.6 ± 2.9	1.51 ± 0.18
	1000	3.35 ± 0.14	40.5 ± 2.5	1.21 ± 0.17
PLA_b1_	0	3.39 ± 0.12	49.0 ± 2.0	1.44 ± 0.29
	500	3.29 ± 0.13	36.9 ± 1.1	1.12 ± 0.04
	1000	3.22 ± 0.08	25.6 ± 2.3	0.80 ± 0.16
PLA_b3_	0	3.13 ± 0.16	32.3 ± 1.9	1.03 ± 0.06
	500	3.01 ± 0.19	24.9 ± 2.5	0.83 ± 0.07
	1000	2.93 ± 0.17	16.1 ± 2.3	0.55 ± 0.09

**Table 5:** Evolution of crack density with exposure time.

Material	Exposure Time (h)	$\phi_{v}\;\left(\%\right)$	ϵ(%)
PLA_s_	0	–	–
	500		1.14
	1000		2.21
PLA_b1_	0	1.24	–
	500		1.78
	1000		3.18
PLA_b3_	0	5.54	–
	500		2.21
	1000		3.93

**Table 6:** Pressure sensitivity coefficient and internal frictional angle of solid matrix.

Material	Exposure Time (h)	Yield Criterion	ω	$\psi_{\omega }\;\left(\deg\right)$
PLA_s_	0	Von Mises	–	–
	500			
	1000			
PLA_b1_	0	Drucker-Prager	0.256	37.5
	500		0.218	33.2
	1000		0.189	29.6
PLA_b3_	0	Drucker-Prager	0.799	67.4
	500		0.551	58.8
	1000		0.364	47.5

**Table 7:** CIELab D65-10 coordinate system for raw PLA and its biocomposites.

Material	Exposure Time (h)	Color Coordinates			Total Coordinate Gradient			
		$L^{*}$	$a^{*}$	$b^{*}$	$\Delta L^{*}$	$\Delta a^{*}$	$\Delta b^{*}$	$\Delta E^{*}$
PLA_s_	0	72.88	−0.02	10.65	0	0	0	0
	500	67.59	−0.06	8.42	−5.29	−0.04	−2.23	5.74
	1000	62.30	−0.13	4.33	−10.58	−0.11	−6.32	12.32
PLA_b1_	0	31.15	2.03	4.44	0	0	0	0
	500	36.51	0.96	1.90	5.36	−1.07	−2.54	6.03
	1000	46.52	−0.25	−1.06	15.37	−2.28	−5.50	16.48
PLA_b2_	0	25.16	0.25	−0.12	0	0	0	0
	500	31.58	−0.05	−0.55	6.42	−0.30	−0.43	6.44
	1000	48.89	−0.24	−0.86	23.73	−0.49	−0.74	23.75

**Table 8:** Specular gloss measurement at 60° for raw PLA and its biocomposites.

Material	Exposure Time (h)	Surface Gloss (GU)
PLA_s_	0	52.5 ± 0.5
	500	38.7 ± 1.4
	1000	25.0 ± 2.2
PLA_b1_	0	47.1 ± 1.8
	500	41.0 ± 1.1
	1000	35.5 ± 0.6
PLA_b3_	0	27.0 ± 1.7
	500	22.9 ± 2.1
	1000	18.8 ± 1.7

**Table 9:** Characteristic FTIR absorption bands of raw PLA and their changes during weathering.

Wavenumber (cm^−1^)	Assignment	Degradation Process
3100–3700	O–H stretching	Hydrolysis (COOH, OH end groups)
2800–3000	C–H stretching (CH, CH_2_, CH_3_)	Backbone structure
1748	C=O stretching (Ester in PLA backbone)	Ester bond cleavage (hydrolysis, photolysis)
1647	C=C stretching (vinyl end groups)	Photolysis (Norrish Type II)
1452	C–H bending/deformation (CH_2)_	Backbone structure
1383	C–H bending/deformation (CH_3_)	Side group structure; thermo-oxidative changes
1358		
1000–1300	C–O stretching (ester C–O–C), C–C stretching	Ester bond cleavage, formation of new end groups, structural changes
955	Skeletal vibrations	Amorphous phase
921	Skeletal vibrations CH_3_ rocking	Crystalline phase contribution (α-crystals)
868	C–C Stretching	Backbone structure

**Table 10:** CI and HI evolution for raw PLA and its biocomposites.

Material	Exposure Time (h)	CI	HI
PLA_s_	0	5.98	0.38
	500	6.04	1.43
	1000	6.12	2.80
PLA_b1_	0	5.99	0.45
	500	5.72	2.11
	1000	4.17	3.19
PLA_b3_	0	5.84	0.49
	500	5.64	2.28
	1000	5.77	5.85

**Table 11:** DSC parameters before and after accelerated weathering (2nd heating).

Material	Exposure Time (h)	$T_{g}$ (°C)	$T_{\mathrm{cc}}$ (°C)	$T_{m}$ (°C)	$\Delta H_{\mathrm{cc}}$ (J/g)	$\Delta H_{m}$ (J/g)	$\chi_{c}$ (%)
PLA_s_	0	49.6	–	172.5	–	35.1	37.5
	500	52.5	–	171.6	–	36.9	39.4
	1000	54.6	–	170.8	–	38.3	40.8
PLA_b1_	0	63.2	109.2	169.2	30.6	42.9	13.3
	500	62.1	109.4	169.0	27.0	43.8	18.1
	1000	60.7	109.5	168.1	25.6	44.4	20.2
PLA_b3_	0	58.4	118.9	161.9	30.3	35.7	6.0
	500	58.0	120.8	158.5	28.4	36.4	8.9
	1000	57.8	120.9	158.1	29.3	38.9	10.5

**Table 12:** Kinetic parameters from model fitting of raw PLA and its composites.

Material	$E_{a}$ (kJ/mol)	$A\times 10^{9}$ ($s^{-1}$)	*n*	RSS	$S^{2}\times 10^{-5}$
PLA_s_	149.8	13.8	1.59	0.33	2.72
PLA_b1_	136.5	8.4	1.12	0.66	5.13
PLA_b3_	122.0	1.7	0.95	0.77	18.83

**Table 13:** Thermal degradation parameters of raw PLA and their biocomposites derived from TGA and DTGA curves.

Material	$T_{\mathrm{onset}}$ (°C) ^1^	$T_{\max}$ (°C) ^2^
PLA_s_	328.5	365.6
PLA_b1_	271.4	319.5
PLA_b3_	234.3	288.4

^1^ Temperature at 5% mass loss. ^2^ Temperature of maximum degradation rate from DTGA.

**Table 14:** Accelerated and outdoor test parameters for the calculation of the AF at a tilt angle of 5°.

Material	H (MJ/m^2^)			T (°C)			RH (%)			AF	
	L.	M.	P.	L.	M.	P.	L.	M.	P.	M.	P.
PLA_s_	1892	340	376	40	30		50	80	34	15	73
PLA_b1_				50	36					22	109
PLA_b3_				60	42					27	135

L. Laboratory test. M. Outdoor test in Miami (estimated). P. Outdoor test in Phoenix (estimated).

## Data Availability

The original contributions presented in this study are included in the article. Further inquiries can be directed to the corresponding author.

## Appendix A. Compressibility P and Shear Q for Prolate Spheroidal Pores

The compressibility *P* and the shear *Q* can be rearranged as closed-form solutions dependent on the averaged aspect ratio γ and the Poisson ratio of the matrix material $\nu_{s}$ as follows:

(A1) $$P=\frac{\left(1-\nu_{s}\right)}{6(1-2\nu_{s})}\frac{4\left(1+\nu_{s}\right)+2\gamma^{2}\left(7-2\nu_{s}\right)-\left(3\left(1+4\nu_{s}\right)+12\gamma^{2}\left(2-\nu_{s}\right)\right)g}{2\gamma^{2}+\left(1-4\gamma^{2}\right)g+\left(\gamma^{2}-1\right)\left(1+\nu_{s}\right)g^{2}}$$

(A2) $$\begin{array}{c} Q=\frac{4\left(\gamma^{2}-1\right)\left(1-\nu_{s}\right)}{15(8\left(\nu_{s}-1\right)+2\gamma^{2}\left(3-4\nu_{s}\right)+\left(\left(7-8\nu_{s}\right)-4\gamma^{2}\left(1-2\nu_{s}\right)\right)g)}\\ (\frac{8\left(1-\nu_{s}\right)+2\gamma^{2}\left(3+4\nu_{s}\right)+\left(\left(8\nu_{s}-1\right)-4\gamma^{2}\left(5+2\nu_{s}\right)\right)g+6\left(\gamma^{2}-1\right)\left(1+\nu_{s}\right)g^{2}}{2\gamma^{2}+\left(1-4\nu_{s}\right)g+\left(\gamma^{2}-1\right)\left(1+\nu_{s}\right)g^{2}}\\ -3\left(\frac{8\left(\nu_{s}-1\right)+2\gamma^{2}\left(5-4\nu_{s}\right)+\left(3\left(1-2\nu_{s}\right)+6\gamma^{2}\left(\nu_{s}-1\right)\right)g}{-2\gamma^{2}+2\left(\left(2-\nu_{s}\right)+\gamma^{2}\left(1+\nu_{s}\right)\right)g}\right)) \end{array}$$

where *g* is a function of γ, which is defined as follows for the prolate spheroids (γ>1):

(A3) $$g=\frac{\gamma }{\sqrt{{\left(\gamma^{2}-1\right)}^{3}}}\left(\gamma \sqrt{\gamma^{2}-1}-{\text{cosh}}^{-1}\left(\gamma \right)\right)$$

## Appendix B. Parameters of the Macroscopic Yield Criterion for Spheroidal Voids

We provide here the expression of *p*, η, ζ, χ, Δ, Υ, τ, Γ, Θ, $\Sigma_{s}$, and $\Sigma_{q}$ as follows:

(A4) $$p^{2}=\frac{3}{2}\frac{\phi_{v}}{\phi_{v}\left(1-\phi_{v}\right)}\left(6\left(\kappa_{1}\left(1-\kappa_{1}\right)-\phi_{v}\kappa_{2}\left(1-\kappa_{2}\right)\right)+2\left(\left(1-\kappa_{1}\right)-\phi_{v}\left(1-\kappa_{2}\right)\right)\right)$$

(A5) $$\eta =\frac{9\phi_{v}\left(\kappa_{2}-\kappa_{1}\right)}{p^{2}\left(1-\phi_{v}\right)}$$

(A6) $$\zeta =\frac{9\phi_{v}{\left(\kappa_{2}-\kappa_{1}\right)}^{2}}{p^{2}\left(1-\phi_{v}\right)}$$

(A7) $$\chi =\frac{4}{3\kappa_{1}-1}$$

(A8) $$\Delta =\frac{1+sgn(\Sigma_{m})}{2}+\left(\frac{1}{1-\zeta }-\eta \right)\frac{1-sgn(\Sigma_{m})}{2}$$

(A9) $$\Upsilon =\frac{\omega }{1-\phi_{v}}$$

(A10) $$\tau =\frac{\Upsilon }{1-\Upsilon \left(3\kappa_{2}-1\right)\frac{\Sigma_{q}}{\sigma_{eff}}}$$

(A11) $$\Gamma =\frac{\left(3\kappa_{2}-1\right)\frac{\Sigma_{q}}{\sigma_{eff}}+\tau {\left(3\kappa_{2}-1\right)}^{2}\frac{\Sigma_{eq}^{2}}{\sigma_{eff}^{2}}+\frac{2\omega +sgn(\Sigma_{m})}{\omega }\ln\left(1-3\omega \frac{\Sigma_{m}}{\sigma_{eff}}\Delta \right)sgn\left(\Sigma_{m}\right)}{\tau \left(3\kappa_{2}-1\right)\left(\Upsilon \left(3\kappa_{2}-1\right)\left(\frac{\eta (3\kappa_{2}-1)}{3}-1\right)\left(\frac{\Sigma_{eq}^{2}}{\sigma_{eff}^{2}}-\frac{\Sigma_{q}^{2}}{\sigma_{eff}^{2}}\right)-\frac{\Sigma_{q}}{\sigma_{eff}}\right)-1}$$

(A12) $$\Theta =1-\Gamma \Upsilon \left(\frac{\eta (3\kappa_{2}-1)}{3}-1\right)$$

(A13) $$\Sigma_{s}=\frac{\Gamma \eta }{3-3\Upsilon \left(3\kappa_{2}-1\right)\frac{\Sigma_{q}}{\sigma_{eff}}}$$

(A14) $$\Sigma_{q}=\Sigma :Q,\;with\;Q=\frac{1}{2}\left(e_{1}⊗e_{1}+e_{2}⊗e_{2}\right)-e_{3}⊗e_{3}$$
